# ActA-mediated PykF acetylation negatively regulates oxidative stress adaptability of *Streptococcus mutans*

**DOI:** 10.1128/mbio.01839-24

**Published:** 2024-09-09

**Authors:** Qizhao Ma, Jing Li, Shuxing Yu, Yaqi Liu, Jing Zhou, Xinyue Wang, Lingyun Wang, Jing Zou, Yuqing Li

**Affiliations:** 1 State Key Laboratory of Oral Diseases, National Clinical Research Center for Oral Diseases, West China Hospital of Stomatology, Sichuan University, Chengdu, China; 2 Department of Pediatric Dentistry, West China Hospital of Stomatology, Sichuan University, Chengdu, China; 3 Section of Infectious Diseases, Department of Internal Medicine, Yale University School of Medicine, New Haven, Connecticut, USA; 4 Center for Archaeological Science, Sichuan University, Chengdu, China; Universite de Geneve, Geneva, Switzerland; University of Florida College of Dentistry, Gainesville, Florida, USA

**Keywords:** *Streptococcus mutans*, protein acetylation, pyruvate kinase, pyruvate, oxidative stress adaptability, interspecies competition

## Abstract

Dental caries is associated with microbial dysbiosis caused by the excessive proliferation of *Streptococcus mutans* in dental biofilms, where oxidative stress serves as the major stressor to microbial communities. The adaptability of *S. mutans* to oxidative stress is a prerequisite for its proliferation and even for exerting its virulence. Protein acetylation is a reversible and conserved regulatory mechanism enabling bacteria to rapidly respond to external environmental stressors. However, the functions of protein acetylation in regulating oxidative stress adaptability of *S. mutans* are still unknown. Here, we unveil the impact of acetyltransferase ActA-mediated acetylation on regulating the oxidative stress response of *S. mutans. actA* overexpression increased the sensitivity of *S. mutans* to hydrogen peroxide and diminished its competitive ability against *Streptococcus sanguinis*. In contrast, *actA* deletion enhanced oxidative stress tolerance and competitiveness of *S. mutans*. The mass spectrometric analysis identified pyruvate kinase (PykF) as a substrate of ActA, with its acetylation impairing its enzymatic activity and reducing pyruvate production. Supplementation with exogenous pyruvate mitigated oxidative stress sensitivity and restored competitiveness in multi-species biofilms. *In vitro* acetylation analysis further confirmed that ActA directly acetylates PykF, negatively affecting its enzymatic activity. Moreover, 18 potential lysine-acetylated sites on PykF were identified *in vitro*, which account for 75% of lysine-acetylated sites detected *in vivo*. Taken together, our study elucidates a novel regulatory mechanism of ActA-mediated acetylation of PykF in modulating oxidative stress adaptability of *S. mutans* by influencing pyruvate production, providing insights into the importance of protein acetylation in microbial environmental adaptability and interspecies interactions within dental biofilms.

**IMPORTANCE:**

Dental caries poses a significant challenge to global oral health, driven by microbial dysbiosis within dental biofilms. The pathogenicity of *Streptococcus mutans*, a major cariogenic bacterium, is closely linked to its ability to adapt to changing environments and cellular stresses. Our investigation into the protein acetylation mechanisms, particularly through the acetyltransferase ActA, reveals a critical pathway by which *S. mutans* modulates its adaptability to oxidative stress, the dominant stressor within dental biofilms. By elucidating how ActA affects the oxidative stress adaptability and competitiveness of *S. mutans* through the regulatory axis of ActA-PykF-pyruvate, our findings provide insights into the dynamic interplay between cariogenic and commensal bacteria within dental biofilms. This work emphasizes the significance of protein acetylation in bacterial stress response and competitiveness, opening avenues for the development of novel strategies to maintain oral microbial balance within dental biofilms.

## INTRODUCTION

Dental caries remains a predominant oral health concern worldwide, largely driven by the dysbiosis in the microbial community of dental biofilms (1, 2). *Streptococcus mutans*, a principal cariogenic bacterium, plays a pivotal role in disrupting the oral microbiota balance through its excessive proliferation in the dental biofilms, thereby creating a cariogenic microenvironment (3, 4). This microenvironment facilitates the transition of certain bacteria into opportunistic pathogens, synergizing with *S. mutans* to potentiate cariogenic effects and culminate in the onset of dental caries. The virulence of *S. mutans*, characterized by its adhesive capabilities, extracellular and intracellular polysaccharide synthesis, biofilm formation, acidogenicity, and aciduricity, has been the focal point of caries research, aiming to attenuate its pathogenicity (5, 6). However, the initial stages of biofilm formation feature a minimal presence of *S. mutans* in a dynamic and multi-species microbial community (7, 8). Therefore, the ability of *S. mutans* to rapidly adapt to the fluctuating environment of biofilms, characterized by nutritional availability, temperature shifts, pH variations, osmotic stress, and oxidative stress, is crucial for its proliferation and cariogenic activity (9–11). Studies on the transcriptome of the plaque microbial community indicate that 50%–75% of gene changes are closely related to oxidative stress, highlighting the significance of adaptation to oxidative stress for bacterial survival (12, 13).

Reactive oxygen species, such as hydrogen peroxide (H_2_O_2_), superoxide anion (O2·^−^), and hydroxyl radical (HO·), are primary inducers of oxidative stress in the biofilms, inflicting damage on bacterial lipids, proteins, and nucleic acids (14). Early colonizing bacteria, such as *Streptococcus gordonii* and *Streptococcus sanguinis*, are associated with lower caries risk (15). These oral commensal streptococci, by producing hydrogen peroxide, inhibit the colonization and growth of cariogenic pathogens in the same ecological niche during the early stages of biofilm formation (16–18). Given that *S. mutans* constitutes only about 2% or even less of the initial bacterial adhesion on the tooth surface, its adaptation to oxidative stress is the premise for its subsequent proliferation and pathogenicity in dental biofilms (19, 20).

Protein acetylation is a reversible and evolutionarily conserved regulatory mechanism involving in diverse cellular processes across prokaryotic and eukaryotic organisms (21, 22). Protein can be covalently acetylated through two mechanisms: enzymatic and nonenzymatic (23). The enzymatic acetylation is conducted by specific acetyltransferase (KAT) that catalyzes the transfer of an acetyl group from acetyl-CoA to the ε-NH_2_ group of lysine residue on substrate proteins (24). The nonenzymatic acetylation is driven by acetyl-CoA or other acetyl donors under certain metabolic conditions (25). Both enzymatic and nonenzymatic acetylation allow the rapid control of protein function efficiently and precisely, enabling bacteria to swiftly respond to changing environmental stimuli without the need for transcription and translational processes (26).

With advances in mass spectrometry (MS)-based proteomics and high-affinity purification of acetylated peptides, more and more acetylated proteins are identified in various types of bacteria, such as *Escherichia coli*, *Salmonella enterica*, *Yersinia pestis*, *Erwinia amylovora*, *Mycobacterium tuberculosis*, *Streptomyces griseus*, and *Porphyromonas gingivalis* (27–33). Our previous study on acetylome profiling in *S. mutans* has revealed that protein acetylation is widely involved in its acidogenicity, biofilm formation, and oxidative stress adaptability (34). Specifically, we have demonstrated that the acetylations of lactate dehydrogenase (Ldh) and glucosyltransferases (Gtfs) attenuate the acidogenic and biofilm-forming abilities of *S. mutans*, respectively (35, 36). However, the specific mechanisms by which protein acetylation regulates the oxidative stress adaptability of *S. mutans* and the acetyltransferase involved remain unclear.

In this study, we discovered that overexpression of acetyltransferase *actA* in *S. mutans* significantly enhanced its sensitivity to hydrogen peroxide stimuli and diminished its competitive capabilities against *S. sanguinis*. Through mass spectrometric analysis, pyruvate kinase (PykF) was identified as a substrate for ActA, with its acetylation negatively impacting the enzymatic activity and subsequently decreasing pyruvate production. The addition of exogenous pyruvate was able to restore the impaired oxidative stress adaptability of the overexpression strain of *actA. In vitro* acetylation analysis further demonstrated that ActA catalyzed the acetylation of PykF in an enzymatic manner. Overall, this study reveals the regulatory mechanism of ActA-mediated acetylation that enables *S. mutans* to adapt to the oxidative challenges in dental biofilms.

## RESULTS

### Impact of *actA* on the oxidative stress adaptability of *S. mutans*

**Fig 1:**
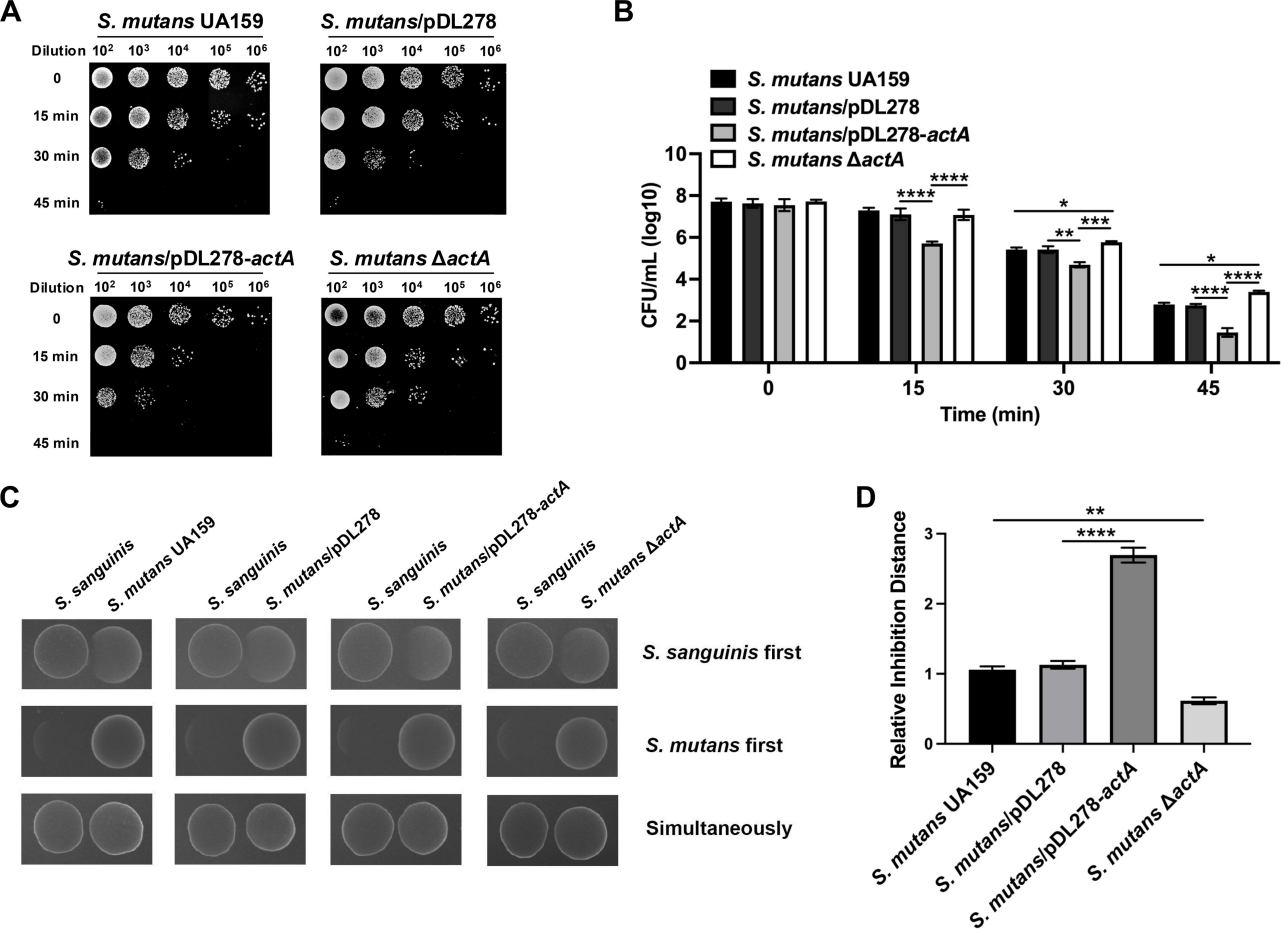
Role of *actA* in oxidative stress adaptability and competitive interactions of *S. mutans*. (**A**) *S. mutans* and its derivatives (OD_600 nm_ = 0.5) were exposed to oxidative stress with 0.2% (66.05 mM) hydrogen peroxide for 0, 15, 30, and 45 min, respectively, which were serially diluted and cultured on the brain heart infusion (BHI) agar plates. The representative pictures of the hydrogen peroxide challenge are shown. (**B**) Colony-forming units (CFUs) were counted for the *S. mutans* and its derivatives post-exposure to hydrogen peroxide at various time intervals. (**C**) Competitive interaction assays between *S. mutans* and *S. sanguinis*. The inhibition patterns of *S. mutans* and its derivatives against *S. sanguinis* are shown when either *S. sanguinis* is inoculated first, *S. mutans* is inoculated first, or both species are inoculated simultaneously. (**D**) Inhibition distances (between the edge of the *S. sanguinis* colony and the *S. mutans* colony) were measured and quantified, shown as relative inhibition distance to *S. mutans*. Results are presented as mean ± SD (*P < 0.05, **P < 0.01, ***P < 0.001, or ****P < 0.0001).

To investigate the role of *actA* in the oxidative stress response of *S. mutans*, we compared the survival rates among the wild-type *S. mutans* UA159, the vector control *S. mutans*/pDL278, the *actA* overexpression strain *S. mutans*/pDL278-*actA*, and the *actA* deletion strain *S. mutans* Δ*actA* following exposure to hydrogen peroxide for intervals of 15, 30, and 45 min (Fig. S1). As shown in Fig. 1A and B, the *actA* overexpression strain *S. mutans*/pDL278-*actA* exhibited significantly reduced survival rates compared to the control *S. mutans*/pDL278, indicating an enhanced sensitivity to hydrogen peroxide-induced oxidative stress. In contrast, the *actA* deletion strain *S. mutans* Δ*actA* exhibited enhanced tolerance to hydrogen peroxide exposure compared to the wild-type *S. mutans* UA159.

As the initial colonizer on the tooth surface, *S. sanguinis* competes with subsequent colonizers *S. mutans* by releasing significant amounts of hydrogen peroxide (37). In contrast, *S. mutans* can resist the oxidative stress caused by hydrogen peroxide through protective mechanisms (38, 39). Therefore, the ability of bacteria to release hydrogen peroxide as well as to deal with its toxicity plays a crucial role in shaping microbial community composition. To investigate the role of *actA* in the adaptability of *S. mutans* to hydrogen peroxide produced by *S. sanguinis*, we conducted interspecies competition analysis, where *S. mutans* and *S. sanguinis* were co-cultured, either sequentially or simultaneously. When *S. mutans* was inoculated prior to *S. sanguinis*, the inhibitory effect of *S. mutans* on *S. sanguinis* was not affected by the presence or absence of *actA* (Fig. 1C, middle row). However, when *S. sanguinis* was inoculated first, its inhibitory effect on the *S. mutans*/pDL278-*actA* strain was enhanced compared to the control *S. mutans*/pDL278, and its inhibitory effect on the *S. mutans* Δ*actA* was diminished compared to the wild-type *S. mutans* UA159 (Fig. 1C and D, upper row). These results indicate that the acetyltransferase ActA plays a significant role in regulating the oxidative stress adaptability of *S. mutans*, impacting its interactions with oral commensal streptococci.

### Identification of PykF as a substrate of acetyltransferase ActA by mass spectrometry

**Fig 2:**
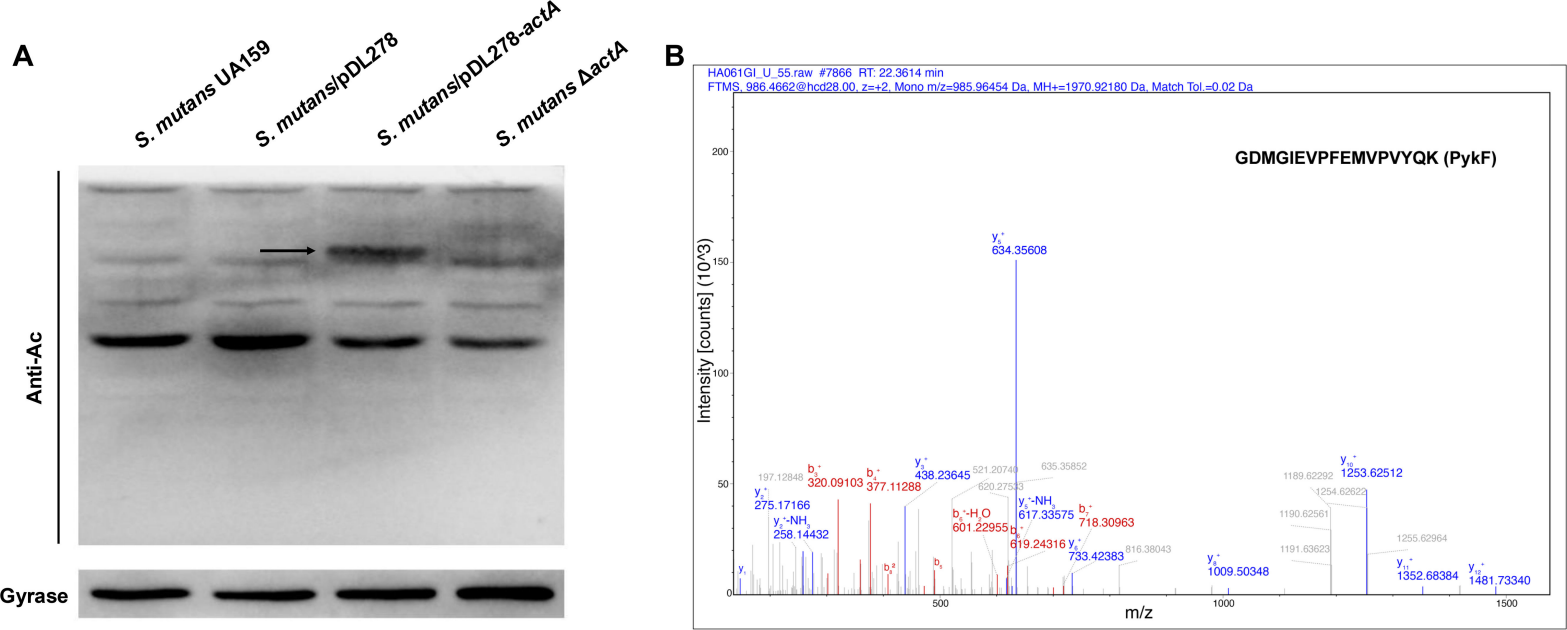
Identification of PykF as a substrate of ActA in *S. mutans*. (**A**) Western blot analysis using anti-acetyl lysine antibodies to detect acetylated proteins in *S. mutans* UA159, *S. mutans*/pDL278, *S. mutans*/pDL278-*actA*, and *S. mutans* Δ*actA*. An increased acetylation signal in the *S. mutans*/pDL278-*actA* strain is marked with an arrow. Gyrase serves as a loading control. (**B**) Mass spectrometry analysis identified the upregulated acetylation band as PykF. The peptide sequence GDMGIEVPFEMVPVYQK from PykF is displayed, along with the mass/charge (m/z) ratios of fragment ions.

To elucidate the potential mechanism by which the acetyltransferase ActA regulates the oxidative stress adaptability of *S. mutans*, we examined the changes in protein acetylation levels among *S. mutans* UA159, *S. mutans*/pDL278, *S. mutans*/pDL278-*actA*, and *S. mutans* Δ*actA*. Utilizing anti-acetyl lysine western blotting analysis, we observed a significantly increased acetylated band in the *S. mutans*/pDL278-*actA* strain, compared to the other three strains (Fig. 2A). Following this observation, we employed MS for unbiased identiﬁcation and found that the increased acetylated band was pyruvate kinase (PykF; Fig. 2B; Table S1). The results suggest that PykF could be the potential substrate of acetyltransferase ActA involved in the oxidative stress adaptability of *S. mutans*.

### Impact of PykF acetylation on the oxidative stress adaptability of *S. mutans*

**Fig 3:**
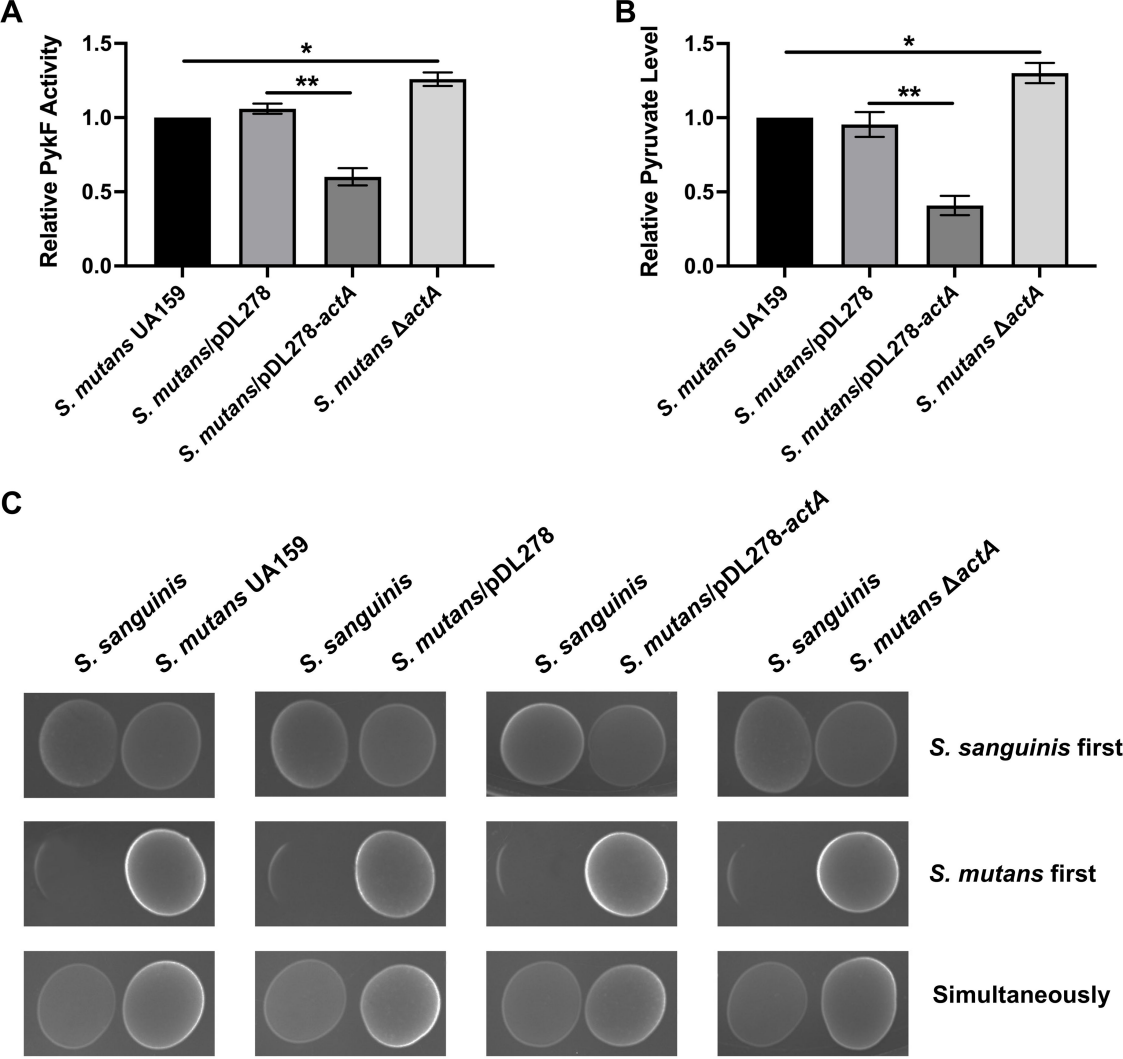
Effect of *actA* on PykF enzymatic activity, pyruvate production, and competitive interactions of *S. mutans* in the presence of sodium pyruvate. (**A**) *S. mutans* and its derivatives were grown to OD_600 nm_ = 0.5, and their total proteins were extracted. The PykF enzymatic activity was measured and normalized to the *S. mutans* UA159. (**B**) The pyruvate levels were measured and normalized to the *S. mutans* UA159. (**C**) Competitive interaction assays between *S. mutans* and *S. sanguinis* after supplementation with exogenous sodium pyruvate. The inhibition patterns of *S. mutans* and its derivatives against *S. sanguinis* are shown when either *S. sanguinis* is inoculated first, *S. mutans* is inoculated first, or both species are inoculated simultaneously. Results are presented as mean ± SD (*P < 0.05 or **P < 0.01).

To delineate how PykF acetylation regulates the oxidative stress adaptability of *S. mutans*, we assessed the enzymatic activity of PykF and the production of its catalytic product, pyruvate. As shown in Fig. 3A and B, *actA* overexpression significantly reduced PykF activity in *S. mutans*, leading to a decreased production of pyruvate. Conversely, *actA* deletion significantly upregulated PykF activity in *S. mutans*, with an increased production of pyruvate (Fig. 3A and B).

To further confirm whether changes in the oxidative stress adaptability of *S. mutans* were attributed to altered pyruvate production, we assessed the impact of exogenous sodium pyruvate on the competitive dynamics between *S. mutans* and the hydrogen peroxide-producing *S. sanguinis*. By supplementing with sodium pyruvate (10 mM), we observed that the inhibitory effect of *S. sanguinis* on *S. mutans* disappeared, regardless of the presence or absence of *actA* (Fig. 3C; Fig. S2). This indicates that the increased inhibitory effect of *S. mutans* on *S. sanguinis* induced by *actA* overexpression was reversed by the addition of exogenous sodium pyruvate (Fig. 1C and 3C). These results show that ActA influences pyruvate production by regulating PykF acetylation and its enzymatic activity, thereby affecting the oxidative adaptability of *S. mutans*.

### *actA* impairs the competitiveness of *S. mutans* in the three-species biofilms

**Fig 4:**
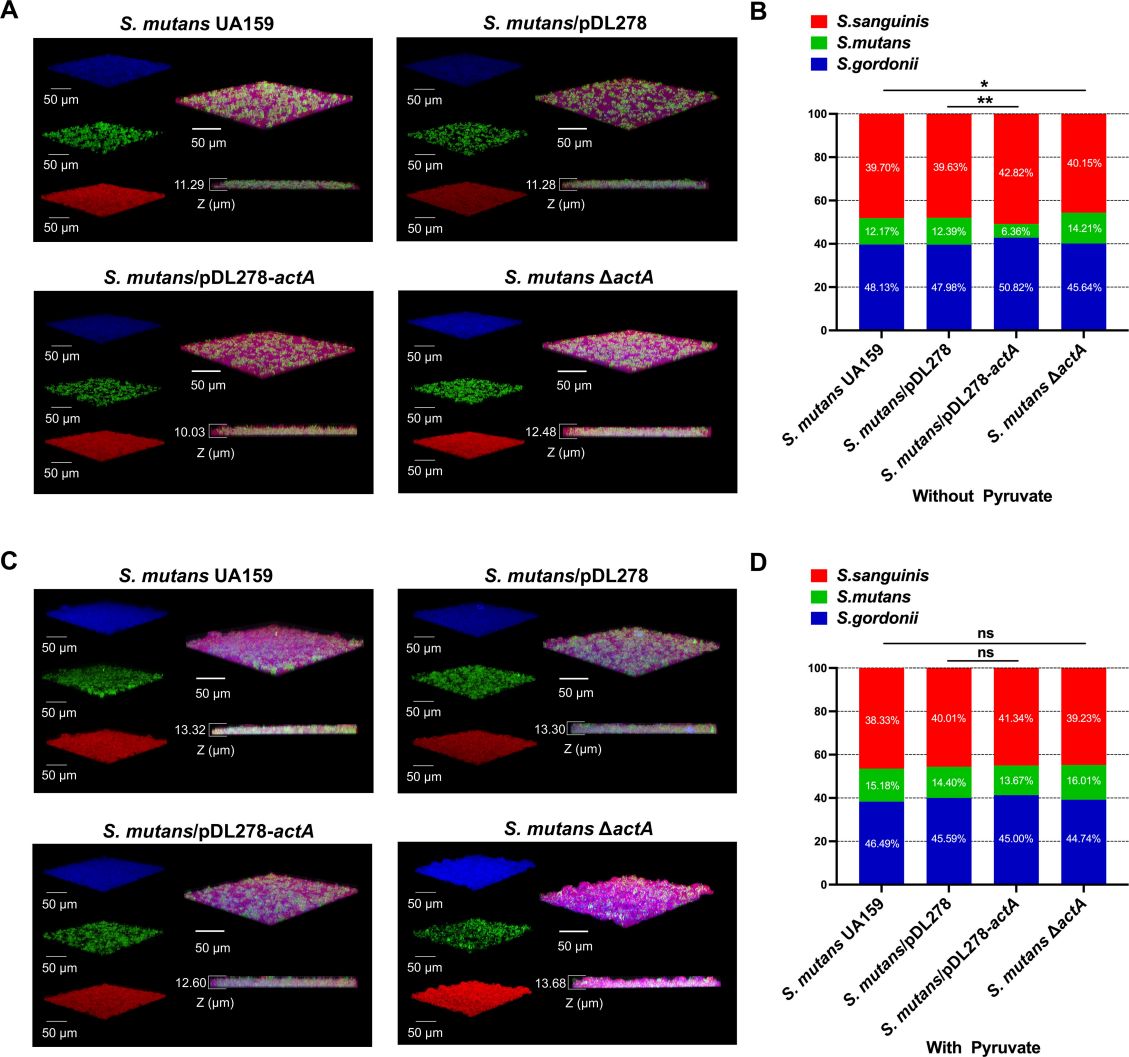
Effect of *actA* on interspecies competition within the three-species biofilms. *S. mutans* or its derivatives, *S. sanguinis*, and *S. gordonii* were simultaneously cultured in fresh BHI supplemented with 1% (wt/vol) sucrose (BHIS) (**A and B**) or supplemented with exogenous sodium pyruvate (**C and D**) under aerobic conditions for 24 h and labeled by species-specific FISH probes. The three-dimensional visualization of the three-species biofilms with *S. mutans* (green), *S. sanguinis* (red), and *S. gordonii* (blue) was captured using confocal laser scanning microscopy at 60× magnification and reconstructed using imaging software IMARIS 7.0.0 (**A and C**). The ratio of *S. mutans*/*S. sanguinis*/*S. gordonii* was quantified by the coverage area of each species using image Pro Plus 6.0 (**B and D**). The representative images are shown from at least five randomly selected positions of each sample. Results are presented as mean ± SD (*P < 0.05 or **P < 0.01, ns: not significant).

*S. sanguinis* and *S. gordonii* are among the first colonizers of the dental surface, and their hydrogen peroxide-dependent antagonism mechanism provides protection against invading caries-associated *S. mutans* (40). The ability of *S. mutans* to contend with and adapt to oxidative stress generated by these commensals is crucial for its competitiveness and survival in the shared ecological niche (41). Therefore, we investigate the influence of the acetyltransferase *actA* on the ability of *S. mutans* to compete with *S. sanguinis* and *S. gordonii* in a three-species biofilm model. Fluorescent *in situ* hybridization (FISH) was used to visualize the spatiotemporal interactions within the three-species biofilm communities (Fig. 4). Quantitative analysis of FISH-labeled biofilms showed that *actA* overexpression significantly diminished the competitive ability of *S. mutans* against *S. sanguinis* and *S. gordonii* (Fig. 4A and B). Conversely, *actA* deletion significantly enhanced the competitiveness of *S. mutans* within three-species biofilms (Fig. 4A and B).

To further probe whether the diminished competitiveness of the overexpression strain *S. mutans*/pDL278-*actA* was due to its reduced production of pyruvate, we supplemented the cultures with sodium pyruvate. As shown in Fig. 4C and D, the addition of exogenous sodium pyruvate significantly reversed the competitive disadvantage of *S. mutans*/pDL278-*actA* against *S. sanguinis* and *S. gordonii* compared to vector control *S. mutans*/pDL278, and no significant competitive differences were observed between *S. mutans* UA159 and *S. mutans* Δ*actA* against *S. sanguinis* and *S. gordonii* within three-species biofilms.

Additionally, we quantified the viable *S. mutans* by counting the colony-forming units (CFUs) in the three-species biofilms, both with and without exogenous sodium pyruvate supplementation. The results showed that the CFU of *S. mutans*/pDL278-*actA* significantly decreased compared to the control *S. mutans*/pDL278. In contrast, the CFU of *S. mutans* Δ*actA* significantly increased compared to the wild-type *S. mutans* UA159 (Fig. S3A). When exogenous sodium pyruvate was added to the three-species biofilms, no significant differences in CFU were observed among *S. mutans* and its derivatives (Fig. S3B). Taken together, these results indicate that the acetyltransferase *actA* regulates the competitiveness of *S. mutans* against oral commensal streptococci by the production of pyruvate within multi-species biofilms.

### ActA mediates PykF acetylation to regulate its enzymatic activity *in vitro*

**Fig 5:**
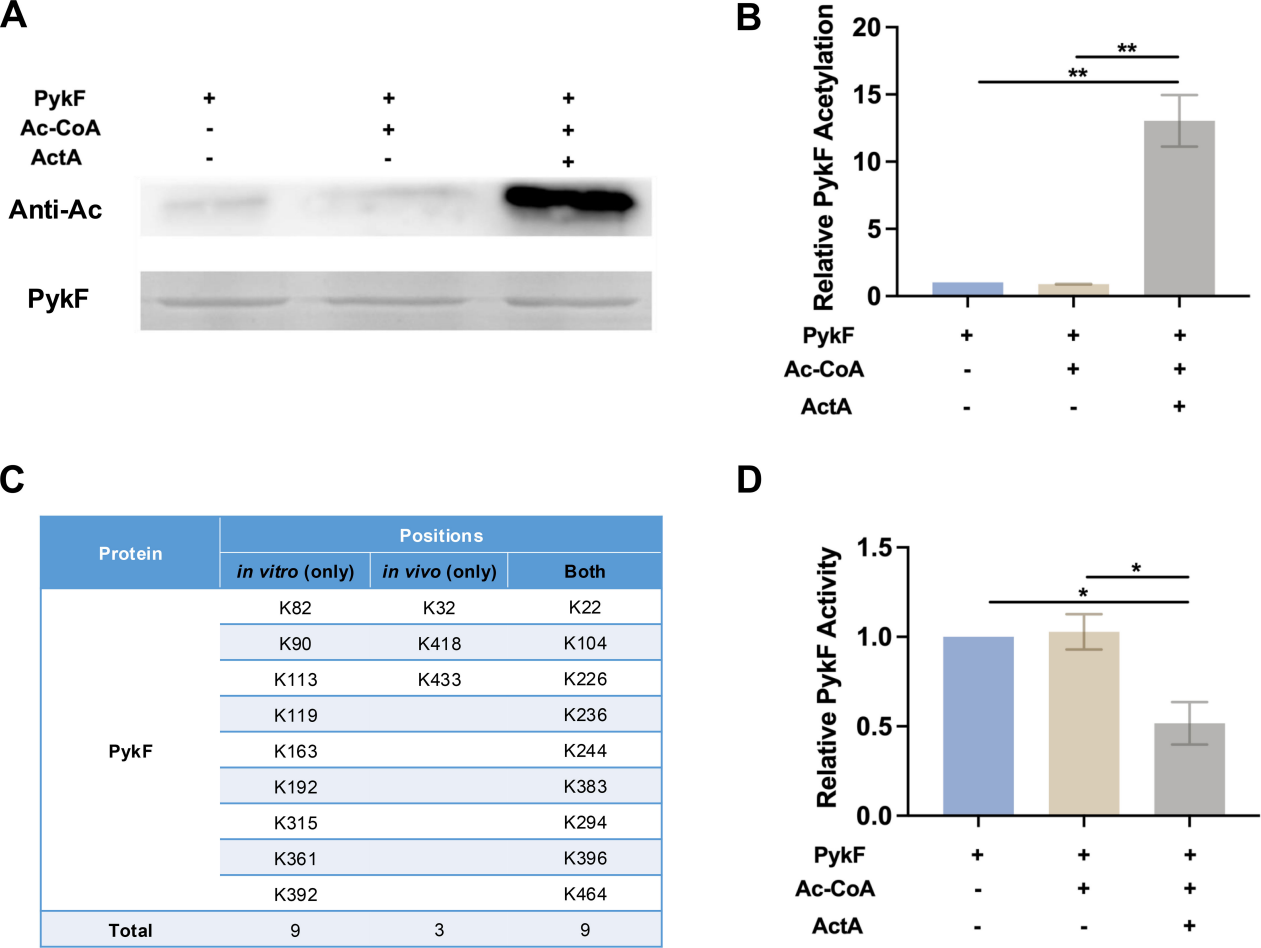
ActA-mediated acetylation of PykF and its effect on enzymatic activity *in vitro*. (**A**) PykF was incubated with or without Ac-CoA in the presence or absence of ActA. Acetylation levels were detected using the anti-acetyl lysine antibody (Anti-Ac). (**B**) The acetylated band signals were quantified using ImageJ software, and then relative acetylation levels were normalized to the control. (**C**) The table lists the acetylated lysine residues identified only *in vitro*, only *in vivo*, and those identified in both conditions, with the total number of acetylated lysine residues in each category provided. (**D**) The enzymatic activity of PykF was measured after incubation with or without Ac-CoA in the presence or absence of ActA and normalized to control. Results are presented as mean ± SD (*P < 0.05 or **P < 0.01).

To further investigate whether ActA could directly acetylate PykF, the *in vitro* acetylation analysis was conducted. First, the *actA* and *pykF* were cloned into expression vectors and purified from corresponding *E. coli* transformants (Fig. S4). Then, the purified ActA was incubated with PykF as the substrate and Ac-CoA as the acetyl donor, and its acetylation level was detected by anti-acetyl lysine western blotting. As shown in Fig. 5A and B, the acetylation of PykF was significantly increased when incubated with ActA in the presence of Ac-CoA compared to the control. Thus, ActA can directly acetylate PykF, which is consistent with the findings shown in Fig. 2.

To identify the specific lysine sites of PykF acetylated by ActA *in vitro*, MS was conducted. The result revealed a total of 18 lysine-acetylated sites on PykF, of which nine sites were consistent with our previous *in vivo* acetylomes findings (Tables S2 and S3). Nine newly lysine-acetylated sites were identified *in vitro*, differing from those reported *in vivo* (Fig. 5C; Table S3). Furthermore, we investigated the effect of the lysine acetylation of PykF on its enzymatic activity. As shown in Fig. 5D, the acetylated PykF negatively regulated its enzymatic activity, which is consistent with the *in vivo* results shown in Fig. 3A. Together, these results demonstrate that ActA directly acetylates PykF and negatively regulates its enzymatic activity.

## DISCUSSION

In dental biofilms, oxidative stress represents a ubiquitous and critical environmental challenge that significantly impacts bacterial survival. Stress response transcriptome reveals that the majority of transcripts (50%–75% of the total) were related to oxidative stress, indicating that oxidative stress may be the dominant stressor to dental biofilm microbial communities (12, 42). *S. mutans* is known as a predominant cariogenic bacterium of dental caries. Its adaptive mechanisms to oxidative stress are essential for bacterial survival and competitiveness, especially within the tight regulated niches (43, 44). Protein acetylation has emerged as a pivotal regulatory mechanism enabling bacteria to rapidly respond to environmental stresses by altering protein stability and activity without the need for transcriptional and translational changes (45). Despite protein acetylation being involved in various biological processes, its role in the oxidative stress adaptability of *S. mutans* remains understudied. Our study bridges this knowledge gap by demonstrating the regulatory mechanisms of the acetyltransferase ActA on the oxidative stress adaptability of *S. mutans*, specifically through modulating the acetylation of PykF, which in turn affects its enzymatic activity and subsequent pyruvate production, a critical metabolite in combating oxidative stress.

In prokaryotes, protein acetylation is modified by acetyltransferase belonging to the GCN5-related N-acetyltransferase (GNAT) superfamily, playing prominent roles across a large number of biological processes including stress response, metabolism, and virulence (46, 47). Our previous study identified that ActA, a GNAT family member, regulates the acidogenicity of *S. mutans* by acetylating lactate dehydrogenase. Given the existence of 15 GNATs and over a thousand acetylated proteins in *S. mutans*, it is plausible that ActA targets multiple substrates, thereby regulating correspondingly biological functions (48). In this study, we extended these findings by using the overexpression strain and deletion strain of *actA*, revealing that *actA* overexpression increases the sensitivity of *S. mutans* to hydrogen peroxide stimuli. Oral commensal streptococci, such as *S. sanguinis*, are among the initial colonizers of the dental surface, capable of producing hydrogen peroxide, which shapes the oxidative stress for subsequent bacterial colonization (49). *S. mutans*, as a later colonizing pathogen, competes with *S. sanguinis*, thus influencing microbial community dynamics (50). This led us to perform the interspecies competition assay, which revealed that *actA* overexpression weakened the competitiveness of *S. mutans* against *S. sanguinis*. These findings underscored the pivotal role of ActA-mediated acetylation in regulating the oxidative stress adaptability of *S. mutans*.

With the advancement of mass spectrometry, an increasing number of substrate proteins undergoing acetylation have been identified (51–53). In our study, we identified PykF as a substrate of ActA based on the observed increased acetylated band and subsequent mass spectrometry analysis. PykF is the key enzyme catalyzing the last step of glycolysis, converting phosphoenolpyruvic acid (PEP) to pyruvate and generating ATP (54). Then, we observed that ActA-mediated acetylation of PykF diminished its enzymatic activity and reduced pyruvate production. Given that pyruvate has been shown to protect bacteria from hydrogen peroxide stress (18, 55), we investigated whether the reduced pyruvate levels were responsible for the decreased oxidative stress adaptability in *actA* overexpression strain *S. mutans*/pDL278-*actA*. As expected, the addition of exogenous sodium pyruvate restored the impaired competitiveness against hydrogen peroxide-producing *S. sanguinis*. This highlights the ability of *S. mutans* to regulate pyruvate production via acetylation modifications, facilitating oxidative adaptability and interspecies competition.

The symbiotic relationship between oral commensal streptococci and the host is integral for the microecological balance within mixed-species dental biofilms (56, 57). The hydrogen peroxide-dependent antagonism exhibited by *S. sanguinis* and *S. gordonii* against cariogenic *S. mutans* underscores the importance of oxidative stress adaptability within the same ecological niche (40, 58). Therefore, the three-species biofilms model was constructed and revealed that *actA* overexpression impairs the competitiveness of *S. mutans* against *S. sanguinis* and *S. gordonii*. This disadvantage was reversed upon supplementation with exogenous sodium pyruvate, emphasizing pyruvate’s critical role in the resistance of *S. mutans* to hydrogen peroxide-producing oral commensal streptococci. Although *actA* overexpression significantly reduced the ratio of *S. mutans* in three-species biofilms, its biological relevance in the multifaceted environment of dental plaque biofilms requires further investigation.

Acetyltransferase catalyzes the transfer of acetyl group from Ac-CoA to lysine residues on substrate proteins, leading to enzymatic acetylation (59). The *in vitro* acetylation analysis provided evidence that ActA can acetylate PykF, thereby influencing its enzymatic activity. In agreement with previous reports, lysine acetylation negatively regulates the catalytic activity of enzymes including topoisomerase I (TopA), acetyl-CoA synthetase, regulators of capsule synthesis B (RcsB), RNase II, and others (60–64). Furthermore, mass spectrometry analysis identified specific lysine acetylation sites on PykF, with discrepancies between the results *in vivo* and *in vitro*, which might arise from differences in cellular context, substrate availability, and the presence of competing or inhibitory factors in the living cell environment.

**Fig 6:**
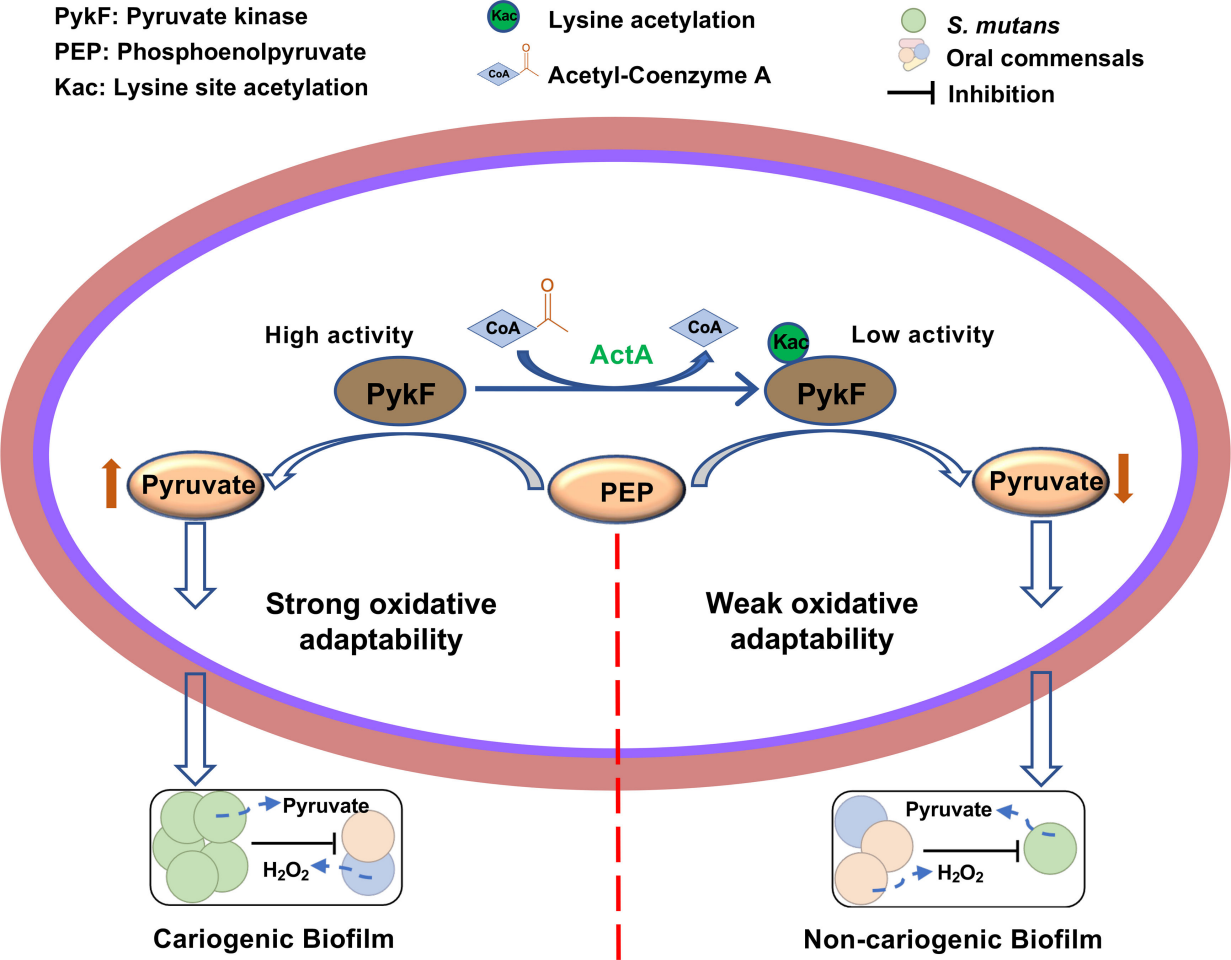
Paradigm of ActA-mediated acetylation of PykF and its impact on oxidative adaptability in *S. mutans*. Non-acetylated PykF with high-enzymatic activity correlates with increased pyruvate production and thereby strong oxidative adaptability and interspecies competitiveness, promoting cariogenic biofilm formation. Acetylation of PykF (indicated by Kac) by ActA reduces its enzymatic activity, leading to decreased pyruvate production and subsequently weakened oxidative adaptability and interspecies competitiveness, associated with non-cariogenic biofilms. The diagram highlights the impact of PykF activity modulated by acetylation on oxidative adaptability and competitive interactions within dental biofilms.

In summary, we originally revealed the regulatory mechanism of acetyltransferase ActA in the oxidative stress response of *S. mutans*. The *in vitro* and *in vivo* analyses demonstrated that ActA influences pyruvate production, a critical metabolite in resistance for hydrogen peroxide, by directly regulating the acetylation of PykF and its enzymatic activity (Fig. 6). In addition, we identified 18 lysine acetylation sites on PykF, which account for 75% of the lysine acetylation sites detected *in vivo*. Further studies should focus on exploring the impact of these specific sites on enzymatic activity and profiling other endogenous candidates of ActA in *S. mutans*. Our study elucidates the acetylation-mediated axis (Acta-PykF-pyruvate) by which *S. mutans* fine-tunes its survival strategy to adapt to oxidative stress within dental biofilms.

## MATERIALS AND METHODS

### Bacterial strains and growth conditions

*S. mutans* UA159, *S. sanguinis* SK1, *S. gordonii* DL1, *E. coli* DH5α, and *E. coli* DL21 (DE3) were used in this study. The *S. mutans*/pDL278 (vector control), *S. mutans*/pDL278-*actA* (*actA* overexpression strain), and *S. mutans* Δ*actA* (*actA* deletion strain) were previously made from the *S. mutans* UA159 background (35). *S. mutans* and its derivatives were cultivated in the brain heart infusion (BHI) broth (Difco, Sparks, MD, USA) at 37°C with 5% CO_2_. *E. coli* and its derivatives were cultivated in Luria-Bertani (LB) broth or on LB agar plates aerobically at 37°C. Antibiotics were used at the following concentrations: spectinomycin (1 mg/mL for *S. mutans* and 100 µg/mL for *E. coli*) and kanamycin (50 µg/mL for *E. coli*) when necessary. All the strains, plasmids, and primers utilized in this study are detailed in Tables S4 and S5.

### RNA extraction and quantitative reverse transcription PCR

Total RNA was extracted from *S. mutans* and its derivatives as previously described (36). First, RNA (1 µg) was reverse-transcribed to cDNA using the cDNA Synthesis Kit (Takara, Shiga, Japan) according to the protocol provided. Quantitative RT-PCR was performed using SYBR Green Master Mix (Thermo Fisher Scientific) on the QuantStudio 6 Flex Real-Time PCR Systems (Thermo Fisher Scientific). Specific primers for the target genes and reference genes are listed in Table S5. The expression levels of target genes were normalized to the reference gene 16S rRNA, and relative expression was calculated using the 2^−ΔΔCT^ method. Each quantitative reverse transcription PCR assay was conducted with three biological replicates.

### Oxidative stress sensitivity assay

*S. mutans* and its derivatives were cultured overnight in BHI medium, followed by a 1:10 dilution and continuous incubation until reaching the optical density of 0.5 at 600 nm (OD_600 nm_). The cultures were then centrifuged at 4,000 g for 10 min at 4°C, and the bacterial pellets were suspended in 0.1 M glycine buffer. Subsequently, the strains were exposed to 0.2% (66.05 mM) hydrogen peroxide for varying durations (0, 15, 30, and 45 min). After exposure, the treated bacterial suspensions were serially diluted, plated on BHI agar plates, and incubated at 37°C for 48 h to assess survival rates. Each oxidative stress sensitivity assay was performed in three biological replicates.

### Interspecies competition assay

The interspecies competition assay was performed following a previous study, with minor modifications (65). Briefly, overnight cultures of *S. mutans* and its derivatives, and *S. sanguinis* SK1, were diluted 1:10 in 1/2 BHI medium and grown until reaching an OD_600 nm_ of 0.5. *S. sanguinis* SK1 was inoculated either prior to or simultaneously with *S. mutans* and its derivatives on 1/2 BHI agar plates with or without sodium pyruvate (10 mM) aerobically. The plates were incubated at 37°C for 48 h, after which the inhibitory effect of *S. sanguinis* on *S. mutans* was evaluated. Each interspecies competition assay was performed in four biological replicates.

### Western blotting

The western blotting was performed as previously described (66). Protein samples were extracted from *S. mutans* and its derivatives using bacterial lysis buffer containing protease inhibitors. Protein concentrations were quantified using the bicinchoninic acid assay (BCA) protein assay kit (Beyotime) according to the manufacturer’s instructions. Equal amounts of protein (30 µg) from each sample were separated on 10% SDS-PAGE and then transferred onto polyvinylidene diﬂuoride membranes (Millipore). The membrane was blocked with 5% non-fat milk in tris-buffered saline (TBS) for 1h at room temperature and incubated with primary antibodies against acetyl-lysine (PTM-105, PTM BIO, Hangzhou, China) and Gyrase (custom-made by HUABIO, Hangzhou, China, as a loading control) overnight at 4°C. Next, the membrane was washed six times with tris-buffered saline with Tween 20 (TBST) and then incubated with horseradish peroxidase (HRP)-conjugated secondary antibodies for 2h at room temperature. The protein bands were visualized using the Bio-Rad GS-700 imaging densitometer following incubation with the UltraSignal ECL Chemiluminescent HRP Substrate kit (4A Biotech, Beijing, China). Quantification of band intensity was performed using ImageJ software. Each experiment was conducted with three biological replicates.

### Liquid chromatography-tandem mass spectrometry (LC-MS/MS) analysis

The identification of PykF and its lysine acetylation were performed using LC-MS/MS analysis. Following SDS-PAGE, targeted gel sections were destained using 50 mM NH_4_HCO_3_ in 50% acetonitrile (vol/vol) until being clear. The gel pieces were then dehydrated with 100 µL of 100% acetonitrile for 5 min, after which the liquid was removed. This was followed by rehydration of the gel pieces in 10 mM dithiothreitol and incubation at 56°C for 1 h. After another dehydration step with 100% acetonitrile, the gel pieces were rehydrated with 55 mM iodoacetamide and incubated at room temperature in the dark for 45 min. The gel pieces were then washed with 50 mM NH_4_HCO_3_ and dehydrated with 100% acetonitrile. Rehydration was performed with 10 ng/µL trypsin in 50 mM NH_4_HCO_3_ on ice for 1 h. Excess liquid was removed, and the gel pieces were digested with trypsin at 37°C overnight. The peptides were extracted first with 50% acetonitrile/5% formic acid and then with 100% acetonitrile. Finally, peptides were completely dried and later resuspended in a 2% acetonitrile with 0.1% formic acid solution.

The tryptic peptides were dissolved in 0.1% formic acid (solvent A) and loaded onto a home-made reversed-phase analytical column (15 cm in length, 75 µm i.d.). A gradient consisting of 6%–23% solvent B (0.1% formic acid in 98% acetonitrile) over 16 min, then from 23% to 35% for 8 min, and finally increasing to 80% for 3 min before holding at 80% for the last 3 min was applied, all at a constant flow rate of 400 nL/min on an EASY-nLC 1000 UPLC system. Peptides were subjected to an nanospray ionization (NSI) source followed by tandem mass spectrometry (MS/MS) in a Q Exactive Plus (Thermo Fisher Scientific) coupled online to the ultra performance liquid chromatography (UPLC). An electrospray voltage of 2.0 kV was applied. The m/z scan range was set from 350 to 1,800 for the full scan, and intact peptides were detected in the Orbitrap at a resolution of 70,000. Peptides were selected for MS/MS using an normalized collision energy (NCE) setting of 28, with fragments detected in the Orbitrap at a resolution of 17,500. A data-dependent procedure alternated between one MS scan followed by 20 MS/MS scans with a 15.0-second dynamic exclusion. The automatic gain control was set to 5E4.

The resulting MS/MS data were processed using Proteome Discoverer 1.3. Tandem mass spectra were searched against the *S. mutans* UA159 protein database. Trypsin/P was specified as the cleavage enzyme, allowing up to two missing cleavages. The mass error was set to 10 ppm for precursor ions and 0.02 Da for fragment ions. Carbamidomethyl on Cys was specified as a fixed modification, and oxidation on Met was specified as a variable modification. Peptide confidence was set to high, with a peptide ion score set at greater than 20.

### PykF activity assay

The enzymatic activity of PykF was assessed using the pyruvate kinase activity assay kit (Solarbio, BC0540), according to the manufacturer’s instructions. Briefly, protein samples were extracted, and their concentrations were determined according to the methods described in the western blotting analysis. The activity of pyruvate kinase was measured by monitoring the conversion of PEP to pyruvate in the presence of ADP, which led to the oxidation of nicotinamide adenine dinucleotide ( (reduced form, NADH) to NAD^+^ in a coupled reaction with lactate dehydrogenase. The decrease in absorbance at 340 nm, indicative of NADH consumption, was measured over time using the spectrophotometer. Each PykF activity assay was performed in three biological replicates.

### Pyruvate production assay

The concentration of pyruvate produced by *S. mutans* and its derivatives was determined using a pyruvate assay kit (Solarbio, BC2205), following the manufacturer’s protocol. Briefly, cell lysates were harvested, and their supernatants were collected after centrifugation to remove cell debris. The supernatant was used for the pyruvate production assay. This assay is based on the reaction between pyruvate and 2,4-dinitrophenylhydrazine, forming pyruvate-2,4-dinitrophenylhydrazone, which exhibits a pink color in an alkaline solution. The amount of pyruvate was quantified by detecting the absorbance change at 520 nm using the spectrophotometer. Each pyruvate production assay was conducted with three biological replicates.

### Purification of recombinant ActA and PykF

The genes encoding *actA* and *pykF* were amplified from *S. mutans* UA159 genomic DNA using PCR and cloned into the pET28a (Novagen) expression vectors. Recombinant plasmids were transformed into *E. coli* BL21 (DE3) cells for protein expression. Transformants were cultured in LB medium supplemented with kanamycin (50 µg/mL) until an OD_600 nm_ of 0.6 was reached, at which point protein expression was induced with 0.5 mM isopropyl *β*-D-1-thiogalactopyranoside. After induction, cells were incubated for 6 h at 37°C to optimize protein expression. Cells were harvested by centrifugation and lysed via sonication. Cell debris was removed by centrifugation, and the supernatant was subjected to affinity chromatography using a nickel-nitrilotriacetic acid agarose resin pre-equilibrated with lysis buffer. Bound proteins were eluted with an imidazole gradient and dialyzed against a buffer containing 20 mM Tris-HCl and 100 mM NaCl, pH 7.5. Purified protein was confirmed by SDS-PAGE. Protein concentrations were determined by the Bradford assay, and aliquots were stored at −80°C until use.

### *In vitro* acetylation assay

For *in vitro* acetylation assays, purified PykF was incubated with or without Ac-CoA and purified recombinant ActA in reaction buffer (100 mM Tris-HCl, 150 mM NaCl, 10 mM MgCl_2_, and pH 8.0) at 37°C for 3 h. The reaction was terminated by the addition of SDS-PAGE loading buffer and heating at 95°C for 10 min. Acetylation levels were analyzed by western blot using an anti-acetyl lysine antibody. In addition, the acetylated bands were analyzed by LC-MS/MS. The *in vitro* acetylation assays were conducted in three biological replicates.

### Bacterial composition analysis in three-species biofilms

Three-species biofilms were constructed and analyzed according to previously established protocols with minor modifications (67). Briefly, overnight cultures of *S. mutans* and its derivatives, *S. sanguinis*, and *S. gordonii* were grown until reaching an OD_600 nm_ of 0.5. These cultures were then simultaneously inoculated at an inoculum ratio of *S. mutans:S. sanguinis:S. gordonii* = 1:1:1 into fresh BHI supplemented with 1% (wt/vol) sucrose with or without exogenous sodium pyruvate under aerobic conditions for 24 h. Next, the three-species biofilms were fixed with 4% paraformaldehyde to preserve their structure for imaging. The fixed biofilms were then subjected to species-specific FISH labeling using oligonucleotide probes that target unique sequences within the 16S rRNA of each bacterial species (Table S5 for details). The probes have been validated by hybridizing them with their respective target strains and checking for specificity and absence of cross-reactivity with non-target strains in other reports (68–70). The fluorescently labeled biofilms were visualized using an Olympus FV3000 confocal laser scanning microscope (Olympus Corporation, Tokyo, Japan) at a magnification of 60×. Images were captured from at least five randomly selected fields for each sample. The captured images were used to reconstruct the three-dimensional structure of the biofilms using the IMARIS software version 7.0.0 (Bitplane, Zurich, Switzerland). Furthermore, the bacterial composition within the biofilms was quantitatively analyzed using Image-Pro Plus software version 6.0 (Media Cybernetics). The bacterial composition analysis in three-species biofilms was performed in three biological replicates.

Following the 24-h incubation, the three-species biofilms were harvested and serially diluted, and aliquots of each dilution were plated on SB-20 agar plates selective for *S. mutans*. The SB-20 agar plates were prepared with the following components per liter: bacto-casitone (15.0 g), yeast extract (5.0 g), L-cysteine (0.2 g), sodium sulfite (0.1 g), sodium acetate (20.0 g), sucrose (200.0 g), agar (15.0 g), distilled water (qsp), and bacitracin added to a final concentration of 0.2 U/mL. Plates were incubated anaerobically at 37°C for 48 h. CFUs were counted and expressed as CFU per milliliter. Each experiment was performed in three biological replicates.

### Statistical analysis

All data were obtained from three independent assays. Data were presented as means ± SD for each group. Statistical analyses were analyzed using Prism 10 (GraphPad Software Inc, San Diego, CA, USA). Statistical significance was determined using one-way analysis of variance (ANOVA) followed by Tukey’s post-hoc test for multiple comparisons. When comparing only two groups, an unpaired two-tailed Student’s *t* test was utilized. A *P*-value of less than 0.05 was considered statistically significant.

## SUPPLEMENTAL MATERIAL

Supplemental material mbio.01839-24-s0001.docx Fig. S1 to S4; Tables S1 to S5.

ASM does not own the copyrights to Supplemental Material that may be linked to, or accessed through, an article. The authors have granted ASM a non-exclusive, world-wide license to publish the Supplemental Material files. Please contact the corresponding author directly for reuse.

## References

[1] Pitts NB, Zero DT, Marsh PD, Ekstrand K, Weintraub JA, Ramos-Gomez F, Tagami J, Twetman S, Tsakos G, Ismail A. 2017. Dental caries. Nat Rev Dis Primers 3:17030. doi:10.1038/nrdp.2017.30 28540937

[2] Peres MA, Macpherson LMD, Weyant RJ, Daly B, Venturelli R, Mathur MR, Listl S, Celeste RK, Guarnizo-Herreño CC, Kearns C, Benzian H, Allison P, Watt RG. 2019. Oral diseases: a global public health challenge. Lancet 394:249–260. doi:10.1016/S0140-6736(19)31146-8 31327369

[3] Koo H, Falsetta ML, Klein MI. 2013. The exopolysaccharide matrix: a virulence determinant of cariogenic biofilm. J Dent Res 92:1065–1073. doi:10.1177/0022034513504218 24045647 PMC3834652

[4] Lin Y, Chen J, Zhou X, Li Y. 2021. Inhibition of Streptococcus mutans biofilm formation by strategies targeting the metabolism of exopolysaccharides. Crit Rev Microbiol 47:667–677. doi:10.1080/1040841X.2021.1915959 33938347

[5] Solbiati J, Frias-Lopez J. 2018. Metatranscriptome of the oral microbiome in health and disease. J Dent Res 97:492–500. doi:10.1177/0022034518761644 29518346 PMC5958373

[6] Bowen WH, Burne RA, Wu H, Koo H. 2018. Oral biofilms: pathogens, matrix, and polymicrobial interactions in microenvironments. Trends Microbiol 26:229–242. doi:10.1016/j.tim.2017.09.008 29097091 PMC5834367

[7] Lemos JA, Palmer SR, Zeng L, Wen ZT, Kajfasz JK, Freires IA, Abranches J, Brady LJ. 2019. The biology of Streptococcus mutans. Microbiol Spectr 7. doi:10.1128/microbiolspec.GPP3-0051-2018 PMC6615571 30657107

[8] Teng F, Yang F, Huang S, Bo C, Xu ZZ, Amir A, Knight R, Ling J, Xu J. 2015. Prediction of early childhood caries via spatial-temporal variations of oral microbiota. Cell Host Microbe 18:296–306. doi:10.1016/j.chom.2015.08.005 26355216

[9] Lemos JAC, Abranches J, Burne RA. 2005. Responses of cariogenic streptococci to environmental stresses. Curr Issues Mol Biol 7:95–107. 15580782

[10] Marx P, Sang Y, Qin H, Wang Q, Guo R, Pfeifer C, Kreth J, Merritt J. 2020. Environmental stress perception activates structural remodeling of extant Streptococcus mutans biofilms. NPJ Biofilms Microbiomes 6:17. doi:10.1038/s41522-020-0128-z 32221309 PMC7101444

[11] Smith EG, Spatafora GA. 2012. Gene regulation in S. mutans: complex control in a complex environment. J Dent Res 91:133–141. doi:10.1177/0022034511415415 21743034

[12] Peterson SN, Meissner T, Su AI, Snesrud E, Ong AC, Schork NJ, Bretz WA. 2014. Functional expression of dental plaque microbiota. Front Cell Infect Microbiol 4:108. doi:10.3389/fcimb.2014.00108 25177549 PMC4132376

[13] Ma M, Eaton JW. 1992. Multicellular oxidant defense in unicellular organisms. Proc Natl Acad Sci U S A 89:7924–7928. doi:10.1073/pnas.89.17.7924 1518815 PMC49827

[14] Yu S, Ma Q, Li Y, Zou J. 2023. Molecular and regulatory mechanisms of oxidative stress adaptation in Streptococcus mutans. Mol Oral Microbiol 38:1–8. doi:10.1111/omi.12388 36088636

[15] Hojo K, Nagaoka S, Ohshima T, Maeda N. 2009. Bacterial interactions in dental biofilm development. J Dent Res 88:982–990. doi:10.1177/0022034509346811 19828884

[16] Baty JJ, Stoner SN, Scoffield JA. 2022. Oral commensal streptococci: gatekeepers of the oral cavity. J Bacteriol 204:e0025722. doi:10.1128/jb.00257-22 36286512 PMC9664950

[17] Redanz S, Cheng X, Giacaman RA, Pfeifer CS, Merritt J, Kreth J. 2018. Live and let die: hydrogen peroxide production by the commensal flora and its role in maintaining a symbiotic microbiome. Mol Oral Microbiol 33:337–352. doi:10.1111/omi.12231 29897662 PMC6158098

[18] Redanz S, Treerat P, Mu R, Redanz U, Zou Z, Koley D, Merritt J, Kreth J. 2020. Pyruvate secretion by oral streptococci modulates hydrogen peroxide dependent antagonism. ISME J 14:1074–1088. doi:10.1038/s41396-020-0592-8 31988475 PMC7174352

[19] Jakubovics NS, Goodman SD, Mashburn‐Warren L, Stafford GP, Cieplik F. 2021. The dental plaque biofilm matrix. Periodontol 2000 86:32–56. doi:10.1111/prd.12361 33690911 PMC9413593

[20] Cheng X, Xu X, Zhou X, Ning J. 2024. Oxidative stress response: a critical factor affecting the ecological competitiveness of Streptococcus mutans J Oral Microbiol 16:2292539. doi:10.1080/20002297.2023.2292539 38405599 PMC10885835

[21] Ren J, Sang Y, Lu J, Yao YF. 2017. Protein acetylation and its role in bacterial virulence. Trends Microbiol 25:768–779. doi:10.1016/j.tim.2017.04.001 28462789

[22] VanDrisse CM, Escalante-Semerena JC. 2019. Protein acetylation in bacteria. Annu Rev Microbiol 73:111–132. doi:10.1146/annurev-micro-020518-115526 31091420 PMC6736716

[23] Macek B, Forchhammer K, Hardouin J, Weber-Ban E, Grangeasse C, Mijakovic I. 2019. Protein post-translational modifications in bacteria. Nat Rev Microbiol 17:651–664. doi:10.1038/s41579-019-0243-0 31485032

[24] Ma Q, Zhang Q, Chen Y, Yu S, Huang J, Liu Y, Gong T, Li Y, Zou J. 2021. Post-translational modifications in oral bacteria and their functional impact. Front Microbiol 12:784923. doi:10.3389/fmicb.2021.784923 34925293 PMC8674579

[25] Lin Y, Ma Q, Yan J, Gong T, Huang J, Chen J, Li J, Qiu Y, Wang X, Lei Z, Zeng J, Wang L, Zhou X, Li Y. 2024. Inhibition of Streptococcus mutans growth and biofilm formation through protein acetylation. Mol Oral Microbiol. doi:10.1111/omi.12452 38224336

[26] Christensen DG, Baumgartner JT, Xie X, Jew KM, Basisty N, Schilling B, Kuhn ML, Wolfe AJ. 2019. Mechanisms, detection, and relevance of protein acetylation in prokaryotes. MBio 10:e02708-18. doi:10.1128/mBio.02708-18 30967470 PMC6456759

[27] Feid SC, Walukiewicz HE, Wang X, Nakayasu ES, Rao CV, Wolfe AJ. 2022. Regulation of translation by lysine acetylation in Escherichia coli. MBio 13:e0122422. doi:10.1128/mbio.01224-22 35604121 PMC9239087

[28] Sun Y, Zhang Y, Zhao T, Luan Y, Wang Y, Yang C, Shen B, Huang X, Li G, Zhao S, Zhao GP, Wang Q. 2023. Acetylation coordinates the crosstalk between carbon metabolism and ammonium assimilation in Salmonella enterica. EMBO J 42:e112333. doi:10.15252/embj.2022112333 37183585 PMC10308350

[29] Paquette N, Conlon J, Sweet C, Rus F, Wilson L, Pereira A, Rosadini CV, Goutagny N, Weber ANR, Lane WS, Shaffer SA, Maniatis S, Fitzgerald KA, Stuart L, Silverman N. 2012. Serine/threonine acetylation of TGFβ-activated kinase (TAK1) by Yersinia pestis YopJ inhibits innate immune signaling. Proc Natl Acad Sci U S A 109:12710–12715. doi:10.1073/pnas.1008203109 22802624 PMC3411953

[30] Wu X, Vellaichamy A, Wang D, Zamdborg L, Kelleher NL, Huber SC, Zhao Y. 2013. Differential lysine acetylation profiles of Erwinia amylovora strains revealed by proteomics. J Proteomics 79:60–71. doi:10.1016/j.jprot.2012.12.001 23234799 PMC4418653

[31] Di Y, Xu S, Chi M, Hu Y, Zhang X, Wang H, Zhang W, Zhang X. 2023. Acetylation of cyclic AMP receptor protein by acetyl phosphate modulates mycobacterial virulence. Microbiol Spectr 11:e0400222. doi:10.1128/spectrum.04002-22 36700638 PMC9927398

[32] Ishigaki Y, Akanuma G, Yoshida M, Horinouchi S, Kosono S, Ohnishi Y. 2017. Protein acetylation involved in streptomycin biosynthesis in Streptomyces griseus. J Proteomics 155:63–72. doi:10.1016/j.jprot.2016.12.006 28034645

[33] Zeng J, Wu L, Chen Q, Wang L, Qiu W, Zheng X, Yin X, Liu J, Ren Y, Li Y. 2020. Comprehensive profiling of protein lysine acetylation and its overlap with lysine succinylation in the Porphyromonas gingivalis fimbriated strain ATCC 33277. Mol Oral Microbiol 35:240–250. doi:10.1111/omi.12312 32939976

[34] Lei L, Zeng J, Wang L, Gong T, Zheng X, Qiu W, Zhang R, Yun L, Yang Y, Li Y. 2021. Quantitative acetylome analysis reveals involvement of glucosyltransferase acetylation in Streptococcus mutans biofilm formation. Environ Microbiol Rep 13:86–97. doi:10.1111/1758-2229.12907 33185947

[35] Ma Q, Pan Y, Chen Y, Yu S, Huang J, Liu Y, Gong T, Zhang Q, Sun Q, Zou J, Li Y. 2022. Acetylation of lactate dehydrogenase negatively regulates the acidogenicity of Streptococcus mutans. MBio 13:e0201322. doi:10.1128/mbio.02013-22 36043788 PMC9600946

[36] Ma Q, Pan Y, Chen Y, Yu S, Huang J, Liu Y, Gong T, Zou J, Li Y. 2021. Acetylation of glucosyltransferases regulates Streptococcus mutans biofilm formation and virulence. PLoS Pathog 17:e1010134. doi:10.1371/journal.ppat.1010134 34860858 PMC8673623

[37] Nobbs A, Kreth J. 2019. Genetics of sanguinis-group streptococci in health and disease. Microbiol Spectr 7. doi:10.1128/microbiolspec.GPP3-0052-2018 PMC11590441 30681069

[38] Hillman JD, Dzuback AL, Andrews SW. 1987. Colonization of the human oral cavity by a Streptococcus mutans mutant producing increased bacteriocin. J Dent Res 66:1092–1094. doi:10.1177/00220345870660060101 3476580

[39] Yan J, Gong T, Ma Q, Zheng T, Chen J, Li J, Jing M, Lin Y, Wang X, Lei L, Wang S, Zeng J, Li Y. 2023. vicR overexpression in Streptococcus mutans causes aggregation and affects interspecies competition. Mol Oral Microbiol 38:224–236. doi:10.1111/omi.12407 36779415

[40] Kreth J, Zhang Y, Herzberg MC. 2008. Streptococcal antagonism in oral biofilms: Streptococcus sanguinis and Streptococcus gordonii interference with Streptococcus mutans. J Bacteriol 190:4632–4640. doi:10.1128/JB.00276-08 18441055 PMC2446780

[41] Zheng L, Itzek A, Chen Z, Kreth J. 2011. Oxygen dependent pyruvate oxidase expression and production in Streptococcus sanguinis. Int J Oral Sci 3:82–89. doi:10.4248/IJOS11030 21485312 PMC3469881

[42] Ciofu O, Moser C, Jensen PØ, Høiby N. 2022. Tolerance and resistance of microbial biofilms. Nat Rev Microbiol 20:621–635. doi:10.1038/s41579-022-00682-4 35115704

[43] Lories B, Roberfroid S, Dieltjens L, De Coster D, Foster KR, Steenackers HP. 2020. Biofilm bacteria use stress responses to detect and respond to competitors. Curr Biol 30:1231–1244. doi:10.1016/j.cub.2020.01.065 32084407 PMC7322538

[44] Džunková M, Martinez-Martinez D, Gardlík R, Behuliak M, Janšáková K, Jiménez N, Vázquez-Castellanos JF, Martí JM, D’Auria G, Bandara HMHN, Latorre A, Celec P, Moya A. 2018. Oxidative stress in the oral cavity is driven by individual-specific bacterial communities. NPJ Biofilms Microbiomes 4:29. doi:10.1038/s41522-018-0072-3 30510769 PMC6258756

[45] Shang S, Liu J, Hua F. 2022. Protein acylation: mechanisms, biological functions and therapeutic targets. Signal Transduct Target Ther 7:396. doi:10.1038/s41392-022-01245-y 36577755 PMC9797573

[46] Burckhardt RM, Escalante-Semerena JC. 2020. Small-molecule acetylation by GCN5-related N-acetyltransferases in bacteria. Microbiol Mol Biol Rev 84:e00090-19. doi:10.1128/MMBR.00090-19 32295819 PMC7160885

[47] Dash A, Modak R. 2021. Protein acetyltransferases mediate bacterial adaptation to a diverse environment. J Bacteriol 203:e0023121. doi:10.1128/JB.00231-21 34251868 PMC8425404

[48] Narita T, Weinert BT, Choudhary C. 2019. Functions and mechanisms of non-histone protein acetylation. Nat Rev Mol Cell Biol 20:156–174. doi:10.1038/s41580-018-0081-3 30467427

[49] Kaspar JR, Lee K, Richard B, Walker AR, Burne RA. 2021. Direct interactions with commensal streptococci modify intercellular communication behaviors of Streptococcus mutans . Isme j 15:473–488. doi:10.1038/s41396-020-00789-7 32999420 PMC8027600

[50] Huang X, Browngardt CM, Jiang M, Ahn SJ, Burne RA, Nascimento MM. 2018. Diversity in antagonistic interactions between commensal oral streptococci and Streptococcus mutans. Caries Res 52:88–101. doi:10.1159/000479091 29258070 PMC5828942

[51] Zee BM, Garcia BA. 2013. Validation of protein acetylation by mass spectrometry. Methods Mol Biol 981:1–11. doi:10.1007/978-1-62703-305-3_1 23381849 PMC4085156

[52] Schilling B, Meyer JG, Wei L, Ott M, Verdin E. 2019. High-resolution mass spectrometry to identify and quantify acetylation protein targets. Methods Mol Biol 1983:3–16. doi:10.1007/978-1-4939-9434-2_1 31087289 PMC6825508

[53] Hansen BK, Gupta R, Baldus L, Lyon D, Narita T, Lammers M, Choudhary C, Weinert BT. 2019. Analysis of human acetylation stoichiometry defines mechanistic constraints on protein regulation. Nat Commun 10:1055. doi:10.1038/s41467-019-09024-0 30837475 PMC6401094

[54] Dong H, Zhang J, Zhang H, Han Y, Lu C, Chen C, Tan X, Wang S, Bai X, Zhai G, Tian S, Zhang T, Cheng Z, Li E, Xu L, Zhang K. 2022. YiaC and CobB regulate lysine lactylation in Escherichia coli. Nat Commun 13:6628. doi:10.1038/s41467-022-34399-y 36333310 PMC9636275

[55] Cheng X, Redanz S, Cullin N, Zhou X, Xu X, Joshi V, Koley D, Merritt J, Kreth J. 2018. Plasticity of the pyruvate node modulates hydrogen peroxide production and acid tolerance in multiple oral streptococci. Appl Environ Microbiol 84:e01697-17. doi:10.1128/AEM.01697-17 29079629 PMC5752870

[56] Zhu L, Kreth J. 2012. The role of hydrogen peroxide in environmental adaptation of oral microbial communities. Oxid Med Cell Longev 2012:717843. doi:10.1155/2012/717843 22848782 PMC3405655

[57] Su Y, Yrastorza JT, Matis M, Cusick J, Zhao S, Wang G, Xie J. 2022. Biofilms: formation, research models, potential targets, and methods for prevention and treatment. Adv Sci (Weinh) 9:e2203291. doi:10.1002/advs.202203291 36031384 PMC9561771

[58] Kolenbrander PE, Palmer RJ, Periasamy S, Jakubovics NS. 2010. Oral multispecies biofilm development and the key role of cell-cell distance. Nat Rev Microbiol 8:471–480. doi:10.1038/nrmicro2381 20514044

[59] Ali I, Conrad RJ, Verdin E, Ott M. 2018. Lysine acetylation goes global: from epigenetics to metabolism and therapeutics. Chem Rev 118:1216–1252. doi:10.1021/acs.chemrev.7b00181 29405707 PMC6609103

[60] VanDrisse CM, Parks AR, Escalante-Semerena JC. 2017. A toxin involved in Salmonella persistence regulates its activity by acetylating its cognate antitoxin, a modification reversed by CobB sirtuin deacetylase. MBio 8:e00708-17. doi:10.1128/mBio.00708-17 28559487 PMC5449658

[61] Lima BP, Thanh Huyen TT, Bäsell K, Becher D, Antelmann H, Wolfe AJ. 2012. Inhibition of acetyl phosphate-dependent transcription by an acetylatable lysine on RNA polymerase. J Biol Chem 287:32147–32160. doi:10.1074/jbc.M112.365502 22829598 PMC3442545

[62] Zhang QF, Gu J, Gong P, Wang XD, Tu S, Bi LJ, Yu ZN, Zhang ZP, Cui ZQ, Wei HP, Tao SC, Zhang XE, Deng JY. 2013. Reversibly acetylated lysine residues play important roles in the enzymatic activity of Escherichia coli N-hydroxyarylamine O-acetyltransferase. FEBS J 280:1966–1979. doi:10.1111/febs.12216 23452042

[63] Song L, Wang G, Malhotra A, Deutscher MP, Liang W. 2016. Reversible acetylation on Lys501 regulates the activity of RNase II. Nucleic Acids Res 44:1979–1988. doi:10.1093/nar/gkw053 26847092 PMC4797298

[64] Zhou Q, Zhou YN, Jin DJ, Tse-Dinh YC. 2017. Deacetylation of topoisomerase I is an important physiological function of E. coli CobB. Nucleic Acids Res 45:5349–5358. doi:10.1093/nar/gkx250 28398568 PMC5605244

[65] Kreth J, Merritt J, Shi W, Qi F. 2005. Competition and coexistence between Streptococcus mutans and Streptococcus sanguinis in the dental biofilm. J Bacteriol 187:7193–7203. doi:10.1128/JB.187.21.7193-7203.2005 16237003 PMC1272965

[66] Ma Q, Gu L, Liao S, Zheng Y, Zhang S, Cao Y, Zhang J, Wang Y. 2019. NG25, a novel inhibitor of TAK1, suppresses KRAS-mutant colorectal cancer growth in vitro and in vivo. Apoptosis 24:83–94. doi:10.1007/s10495-018-1498-z 30515612

[67] Yu S, Ma Q, Huang J, Liu Y, Li J, Wang Y, Gong T, Zhang Q, Zou J, Li Y. 2024. SMU_1361c regulates the oxidative stress response of Streptococcus mutans. Appl Environ Microbiol 90:e0187123. doi:10.1128/aem.01871-23 38299814 PMC10880606

[68] Ren Z, Jeckel H, Simon-Soro A, Xiang Z, Liu Y, Cavalcanti IM, Xiao J, Tin NN, Hara A, Drescher K, Koo H. 2022. Interkingdom assemblages in human saliva display group-level surface mobility and disease-promoting emergent functions. Proc Natl Acad Sci U S A 119:e2209699119. doi:10.1073/pnas.2209699119 36191236 PMC9565521

[69] Zhang K, Wang S, Zhou X, Xu HHK, Weir MD, Ge Y, Li M, Wang S, Li Y, Xu X, Zheng L, Cheng L. 2015. Effect of antibacterial dental adhesive on multispecies biofilms formation. J Dent Res 94:622–629. doi:10.1177/0022034515571416 25715378 PMC4485219

[70] Klug B, Rodler C, Koller M, Wimmer G, Kessler HH, Grube M, Santigli E. 2011. Oral biofilm analysis of palatal expanders by fluorescence in-situ hybridization and confocal laser scanning microscopy. JoVE. doi:10.3791/2967 PMC3227208 22041974

