# Supplementary material for: ActA-mediated PykF acetylation negatively regulates oxidative stress adaptability of Streptococcus mutans
Source: mBio. 2024 Sep 9;15(10):e01839-24. doi: 10.1128/mbio.01839-24 (PMC11481489; doi:10.1128/mbio.01839-24)
Supplement: Supplemental material — Fig. S1 to S4; Tables S1 to S5. [file mbio.01839-24-s0001.docx]

**SUPPLEMENTAL MATERIALS FOR**

**Title: ActA-mediated PykF acetylation negatively regulates oxidative stress adaptability of *Streptococcus mutans***

Qizhao Ma^1, 2^, Jing Li^1, 2^, Shuxing Yu^1, 2^, Yaqi Liu^1, 2^, Jing Zhou^1, 2^, Xinyue Wang^1, 2^, Lingyun Wang^3^, Jing Zou^1, 2*^, Yuqing Li^1,^ ^4*^

^1^ State Key Laboratory of Oral Diseases, National Clinical Research Center for Oral Diseases, West China Hospital of Stomatology, Sichuan University, Chengdu, China

^2^ Department of Pediatric Dentistry, West China Hospital of Stomatology, Sichuan University, Chengdu, China

^3^ Section of Infectious Diseases, Department of Internal Medicine, Yale University School of Medicine, New Haven, United States

^4^ Center for Archaeological Science, Sichuan University, Chengdu, China

Running title: ActA-mediated PykF acetylation in *S. mutans*

* Correspondence to:

Email: [zoujing@scu.edu.cn](mailto:zoujing@scu.edu.cn) (JZ), [liyuqing@scu.edu.cn](mailto:liyuqing@scu.edu.cn) (YL)

**FIGURES AND FIGURE LEGENDS**

**
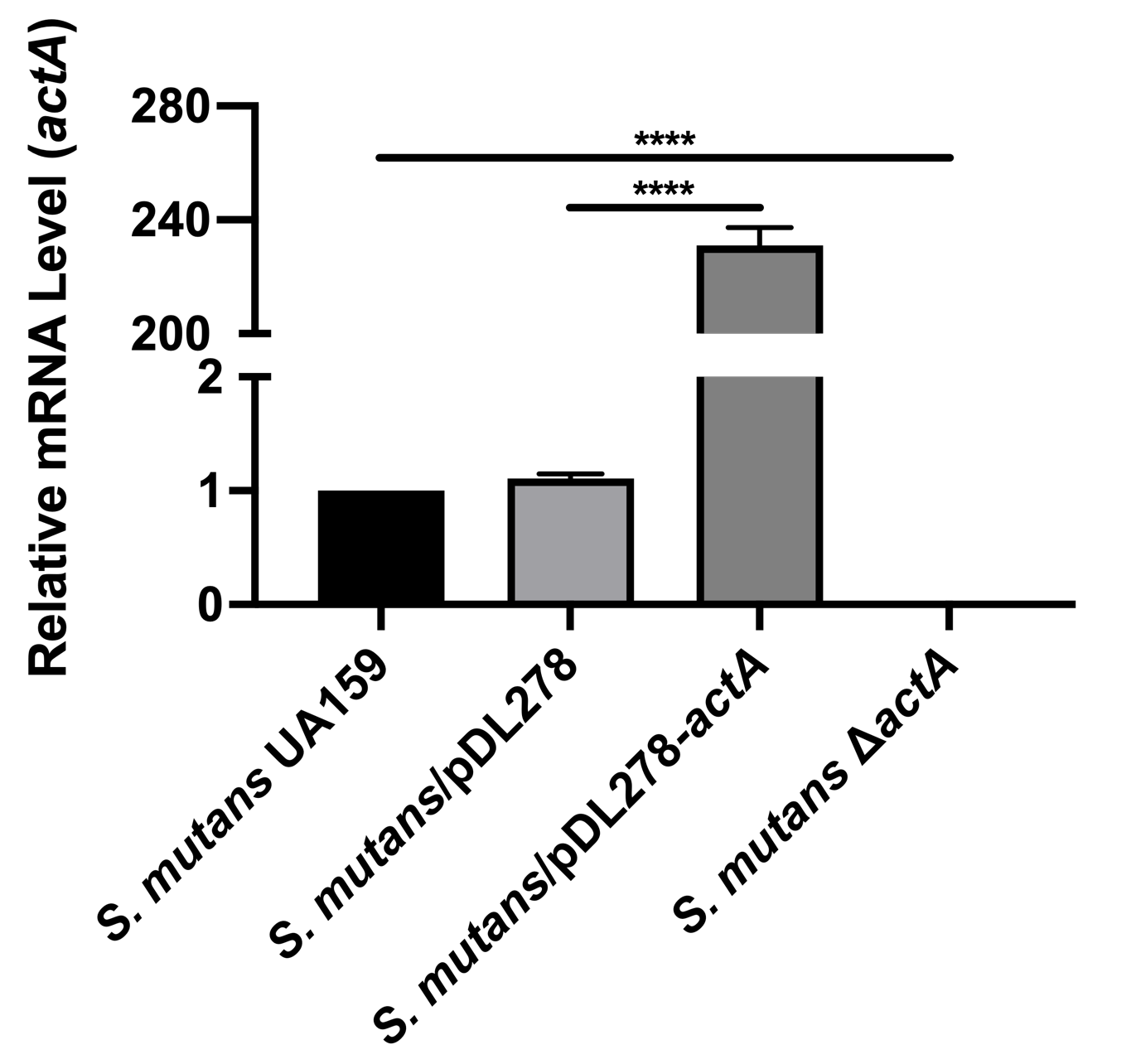
**

**FIG S1 Relative expression levels of *actA* mRNA in *S. mutans* and its derivatives.** Total RNA was extracted from *S. mutans* UA159, *S. mutans*/pDL278, *S. mutans*/pDL278-*actA*, and *S. mutan* Δ*actA* strains*.* The *actA* mRNA levels were determined by quantitative RT-PCR and calculated using the 2^-ΔΔCt method, with values normalized to the reference gene 16S rRNA. The results are presented as mean ± SD (**** *p* <0.0001).


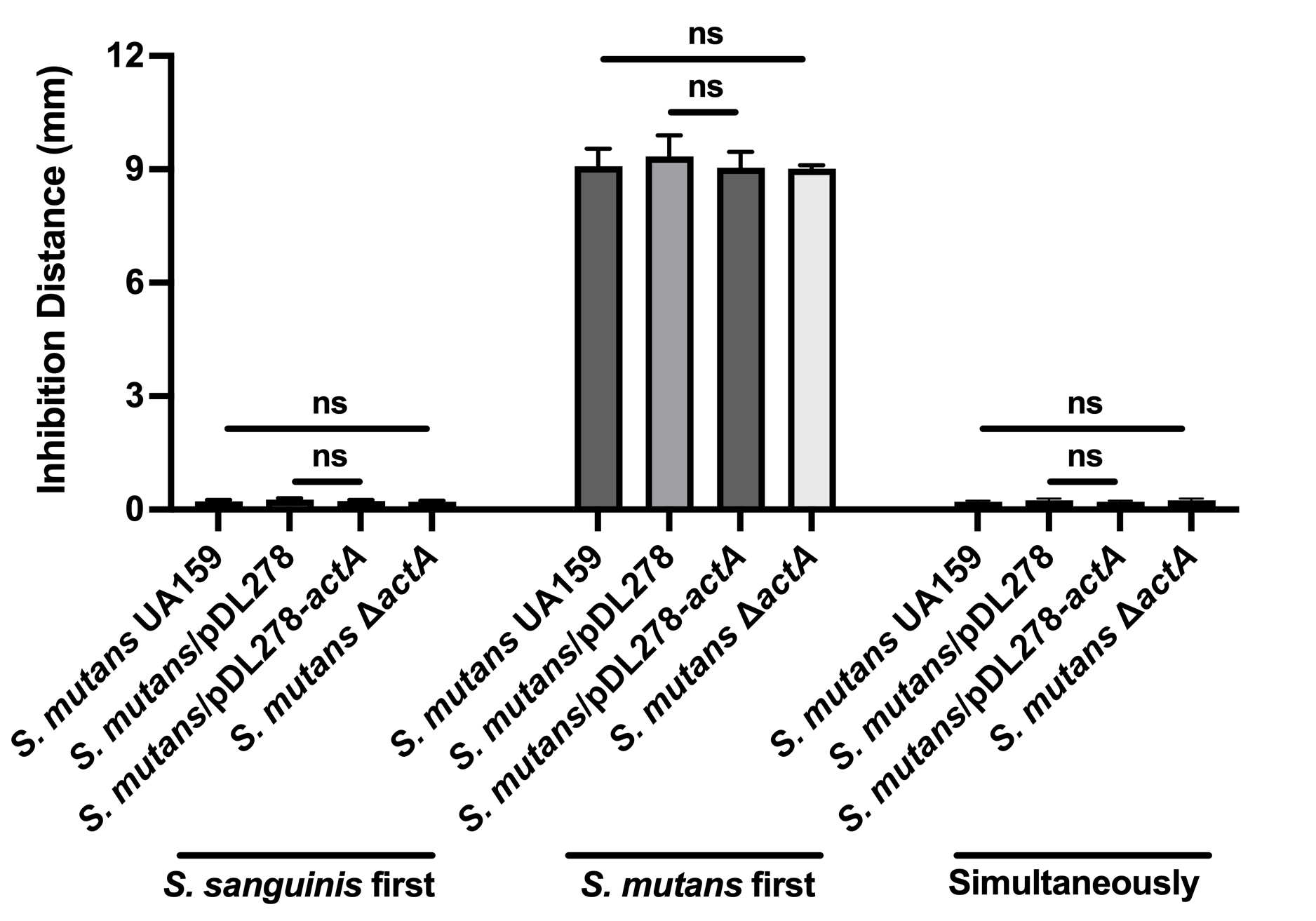


**FIG S2 Quantitative analysis of competitive interactions between *S. mutans* and *S. sanguinis* in the presence of sodium pyruvate.** Inhibition Distances (between the edge of the *S. mutans* colony and the *S. sanguinis* colony) were measured and quantified after supplementation with exogenous sodium pyruvate (10 mM). Results are presented as mean ± SD (ns: not significant).


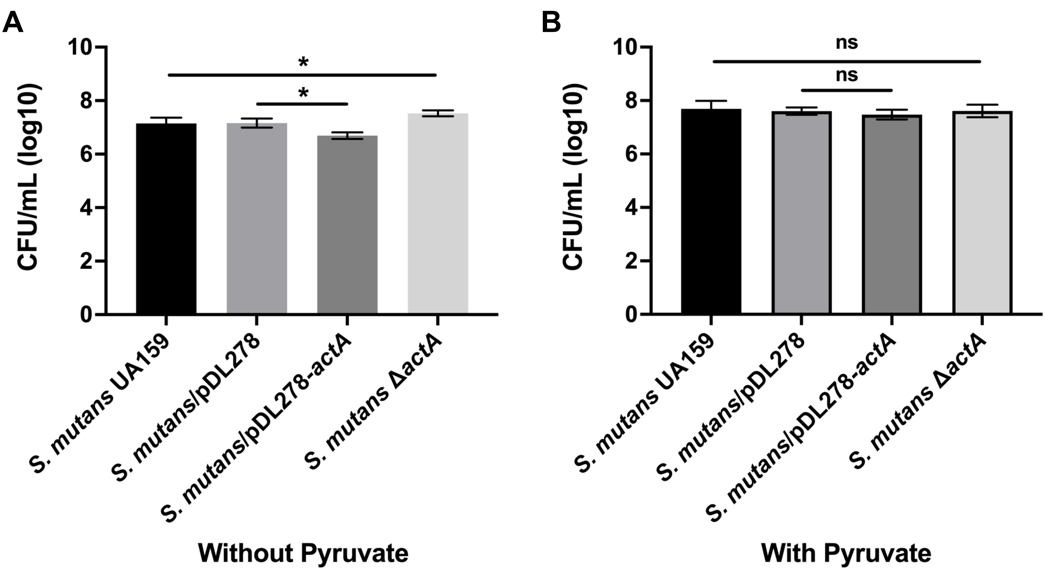


**FIG S3 Quantification of viable *S. mutans* colony forming units (CFU) in the** **three-species biofilms.** *S. mutans* or its derivatives, *S. sanguinis*, and *S. gordonii* were simultaneously cultured in fresh BHIS (A) or supplemented with exogenous sodium pyruvate (B) under aerobic conditions for 24 h. Then, the three-species biofilms were harvested, serially diluted, and plated on selective SB-20 agar for 48 h at 37°C to quantify the viable *S. mutans* CFU. Results are presented as mean ± SD (* *p* < 0.05, ns: not significant).


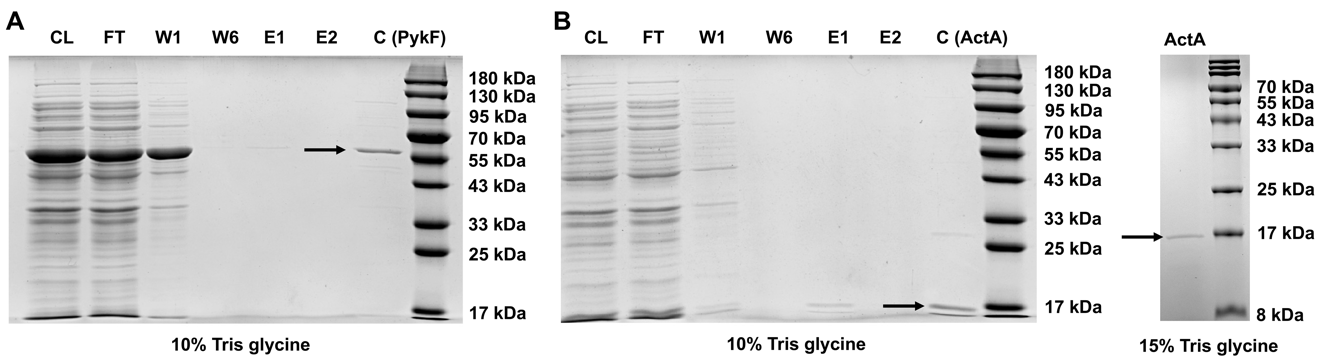


**FIG S4 Purification diagram of recombinant protein of PykF and ActA by SDS-PAGE.** The lanes are as follows: CL (Cell Lysate), FT (Flow Through), W1 (Wash 1), W6 (Wash 6), E1 (Elution 1), E2 (Elution 2), and C (Concentration), with the concentrated PykF (A) and ActA (B) indicated by a black arrow. In panel B, a 15% Tris glycine gel was used to better resolve the 17 kDa ActA protein band, as highlighted by the black arrow. Molecular weight markers are labeled on the right side of each panel.

**TABLES**

**TABLE S1** Identified target substrate (PykF) of ActA by MS analysis.

| **Accession** | **Protein names** | **Gene names** | **MW [kDa]** | **Protein score** | **Sequence coverage (%)** | **Unique Peptides** |
| --- | --- | --- | --- | --- | --- | --- |
| Q8DTX7 | Pyruvate kinase | *pykF* | 54.33 | 3733.58 | 76.13 | 32 |
| Q8DWH9 | Putative threonine synthase | *thrC* | 53.92 | 1207.62 | 54.50 | 23 |
| Q8DTC6 | Phosphoglucosamine mutase | *glmM* | 48.68 | 1204.48 | 51.68 | 20 |
| Q8DSG5 | Glutamyl-tRNA(Gln) amidotransferase subunit A | *gatA* | 52.86 | 1145.91 | 48.25 | 20 |
| Q8DSG6 | Aspartyl/glutamyl-tRNA(Asn/Gln) amidotransferase subunit B | *gatB* | 53.47 | 841.89 | 45.69 | 22 |
| Q8DTM2 | Asparagine--tRNA ligase | *asnS* | 51.02 | 815.07 | 38.84 | 19 |
| Q8DTX7 | Pyruvate kinase | *pykF* | 54.33 | 791.94 | 42.80 | 18 |
| Q8DVE0 | Cell division protein FtsA | *ftsA* | 49.07 | 780.32 | 50.99 | 17 |
| Q8CWW6 | 60 kDa chaperonin | *groL* | 57.07 | 741.29 | 40.96 | 19 |
| Q8DSB7 | Serine--tRNA ligase | *serS* | 48.36 | 715.07 | 47.18 | 15 |
| Q8DUX4 | Putative protease | *smu_761* | 47.88 | 644.34 | 37.62 | 11 |

**TABLE S2** Lysine acetylation sites of PykF identified by LC-MS/MS *in vitro* acetylation analysis.

| **Protein**  **accession** | **Protein names** | **Protein score** | **Sequence coverage (%)** | **Position** | **Peptide score** | **Modified sequence** |
| --- | --- | --- | --- | --- | --- | --- |
| Q8DTX7 | PykF | 4182.36 | 87.2 | K 361 | 80.23 | TmATIDK(Ac)*NAQTLLNEYGR |
| Q8DTX7 | PykF | 4182.36 | 87.2 | K 315 | 70.86 | SAITATNmLETmTDK(Ac)*PR |
| Q8DTX7 | PykF | 4182.36 | 87.2 | K 383^a^ | 67.77 | TNK(Ac)*TEVVASAVK |
| Q8DTX7 | PykF | 4182.36 | 87.2 | K 294^a^ | 61.74 | mIITK(Ac)*VNAAGK |
| Q8DTX7 | PykF | 4182.36 | 87.2 | K 392 | 44.61 | TEVVASAVK(Ac)*DATK |
| Q8DTX7 | PykF | 4182.36 | 87.2 | K 226^a^ | 44.56 | TAK(Ac)*DVNEVR |
| Q8DTX7 | PykF | 4182.36 | 87.2 | K 119 | 42.51 | IATK(Ac)*QGIESTK |
| Q8DTX7 | PykF | 4182.36 | 87.2 | K 163 | 41.76 | LGLTVTAK(Ac)*DITTR |
| Q8DTX7 | PykF | 4182.36 | 87.2 | K 104^a^ | 37.45 | TELFADGVK(Ac)*EYEYK |
| Q8DTX7 | PykF | 4182.36 | 87.2 | K 192 | 36.45 | GVNIPNTK(Ac)*IPFPALAER |
| Q8DTX7 | PykF | 4182.36 | 87.2 | K236^a^ | 33.31 | QICK(Ac)*ETGNEHVK |
| Q8DTX7 | PykF | 4182.36 | 87.2 | K 22^a^ | 32.79 | K(Ac)*FGEDGYWGEK |
| Q8DTX7 | PykF | 4182.36 | 87.2 | K 113 | 31.28 | TGDK(Ac)*LR |
| Q8DTX7 | PykF | 4182.36 | 87.2 | K 90 | 31.26 | VGFLLDTK(Ac)*GPEmR |
| Q8DTX7 | PykF | 4182.36 | 87.2 | K 396^a^ | 24.99 | DATK(Ac)*SmDIK |
| Q8DTX7 | PykF | 4182.36 | 87.2 | K 82 | 24.18 | QK(Ac)*VGFLLDTK |
| Q8DTX7 | PykF | 4182.36 | 87.2 | K 244^a^ | 21.88 | ETGNEHVK(Ac)*LFAK |
| Q8DTX7 | PykF | 4182.36 | 87.2 | K 464^a^ | 21.14 | PASTDDmFEIAEK(Ac)*AALK |

* Indicating lysine acetylation sites of PykF identified *in vitro* acetylation analysis.

^a^ Indicating lysine acetylation sites of PykF also identified *in vivo*.

**TABLE S3** Lysine acetylation sites of PykF identified in the acetylome profiles of *S. mutans*.

| **Protein accession** | **Gene names** | **Position** | **Peptide score** | **Modified sequence** | **Reference** |
| --- | --- | --- | --- | --- | --- |
| Q8DTX7 | *pykF* | K 418 | 142.85 | LISK(Ac)YRPDADILALTFDEK | (1) |
| Q8DTX7 | *pykF* | K 244 | 158.31 | ETGNEHVK(Ac)LFAK |  |
| Q8DTX7 | *pykF* | K 294 | 182.64 | MIITK(Ac)*VNAAGK |  |
| Q8DTX7 | *pykF* | K 32 | 159.78 | KFGEDGYWGEK(Ac)*LDVEASAAK |  |
| Q8DTX7 | *pykF* | K 433 | 174.02 | YRPDADILALTFDEK(Ac)*VQK |  |
| Q8DTX7 | *pykF* | K 396 | 148.32 | DATK(Ac)*SMDIK |  |
| Q8DTX7 | *pykF* | K 104 | 212.95 | TELFADGVK(Ac)*EYEYK |  |
| Q8DTX7 | *pykF* | K 236 | 142.38 | QICK(Ac)*ETGNEHVK |  |
| Q8DTX7 | *pykF* | K 22 | 127.17 | K(Ac)*FGEDGYWGEK |  |
| Q8DTX7 | *pykF* | K 464 | 58.159 | PASTDDMFEIAEK(Ac)*AALK |  |
| Q8DTX7 | *pykF* | K 226 | 114.4 | TAK(Ac)*DVNEVR |  |
| Q8DTX7 | *pykF* | K383 | 172.99 | TNK(Ac)*TEVVASAVK |  |

* Indicating lysine acetylation sites of PykF identified in the acetylome profiles of *S. mutans*.

**TABLE S4** Bacterial strains and plasmids used in this study.

| **Strains or plasmids** | **Description** | **Source** |
| --- | --- | --- |
| ***S. mutans*** |  |  |
| UA159 | Wild type strain | ATCC 700610 |
| *S. mutans*/pDL278 | UA159/pDL278; Spe^r^ | This study |
| *S. mutans*/pDL278*-actA* | UA159/pDL278*-386; Spe^r^* | This study |
| *S. mutan* Δ*actA* | UA159 Δ*386*; Em^s^; p-Cl-Phe^r^ | This study |
| ***E. coli*** |  |  |
| DH5α | F- φ80dlacZΔM15 Δ(lacZYA-argF)U169 deoR recA1 endA1 hsdR17 | Laboratory stock |
|  | (rk-，mk+) phoA supE44 λ- thi-1 gyrA96 relA1 |  |
| BL21(DE3) | F- ompT hsdS B(rB-mB-)dcm gal (DE3) | Novagen |
| **Plasmids** |  |  |
| pET28a | Kan^r^ expression vector with the 6His-tag coding sequence | Laboratory stock |
| pET*actA* | pET derivative for expression 6His-ActA | This study |
| pET*pykF* | pET derivative for expression 6His-PykF | This study |
| pDL278 | *E. coli*-*Streptococcus* shuttle vector (Spe^r^) | Laboratory stock |
| pDL278*-actA* | pDL278 derivative for overexpression of *smu_386* in *S. mutans* | This study |

**TABLE S5** Primers used in this study.

| **Primers** | **Sequence (5’ to 3’)** | **Used in** |
| --- | --- | --- |
| pDL278 F | GTCGACCTGCAGGCATGC | Overexpression |
| pDL278 R | GAGCTCGAATTCACTGGCCG | Overexpression |
| *actA* LDHF | CGGCCAGTGAATTCGAGCTCAGAGCCCGAGCAACAATAACACT | Overexpression |
| *actA* LDHR | CTTTCATCATGTTCTAAACATCTCCTTATAATTT | Overexpression |
| *actA* overF | TGTTTAGAACATGATGAAAGATGAGAAAAACCAAG | Overexpression |
| *actA* overR | TTGCATGCCTGCAGGTCGACTTAATAATCTATTAAACCTTCACGT | Overexpression |
| *actA* upF | CCTAGCAGATTGGTACAG | In-frame deletion construction |
| *actA* upR | GAGTGTTATTGTTGCTCGGCATTACACCCGTTTGACA | In-frame deletion construction |
| *actA* dnF | GGTATACTACTGACAGCTTCGATAAGAAAGACATATGATAG | In-frame deletion construction |
| *actA* dnR | CATATTCAACGGCCGCAG | In-frame deletion construction |
| *actA* checkF | AATCTACTCATCTGTGAAGAG | In-frame deletion construction |
| *actA* checkR | TAGCATTTAGATCGAGTTCTG | In-frame deletion construction |
| *actA* updnR | CTATCATATGTCTTTCTTATCCATTACACCCGTTTGACA | In-frame deletion construction |
| ActA protein F | AAATTTGAATTCATATGATGAAAGATGAGAAAAACCA | Protein expression |
| ActA protein R | ATATATCTCGAGTTAATAATCTATTAAACCTTCAC | Protein expression |
| PykF protein F | CCCAAAGCGGCCGCTATGAATAAACGCGTAAAAATTGTTG | Protein expression |
| PykF protein R | CCCAAACTCGAGTTATTGAACGGTACGAACACGCATAGTATTG | Protein expression |
| *Ldh* F | CCGAGCAACAATAACACTC | In-frame deletion construction |
| *Erm* R | GAAGCTGTCAGTAGTATACC | In-frame deletion construction |
| *16S* F | CCATGTGTAGCGGTGAAATGC | qRT-PCR |
| *16S* R | TCATCGTTTACGGCGTGGAC | qRT-PCR |
| *actA* F | ATCAAGGACAGGGGCTAGGT | qRT-PCR |
| *actA* R | TTGTGCTGCTTGATTGGAAG | qRT-PCR |
| *S. mutans* | ACTCCAGACTTTCCTGAC | FISH |
| *S. sanguinis* | GCATACTATGGTTAAGCCACAGCC | FISH |
| *S. gordonii* | ACTGTGCGTTCTACTTGC | FISH |

**References**

1 Lei, L. *et al.* Quantitative acetylome analysis reveals involvement of glucosyltransferase acetylation in Streptococcus mutans biofilm formation. *Environmental Microbiology Reports* **13**, 86-97 (2020). <https://doi.org/10.1111/1758-2229.12907>
